# Enhancing Therapeutic Efficacy in Cancer Treatment: Integrating Nanomedicine with Autophagy Inhibition Strategies

**DOI:** 10.1021/acsomega.4c02234

**Published:** 2024-06-18

**Authors:** Nada Walweel, Omer Aydin

**Affiliations:** † Department of Biomedical Engineering, Erciyes University, Kayseri 38039, Turkey; ‡ NanoThera Lab, ERFARMA-Drug Application and Research Center, Erciyes University, Kayseri 38280, Turkey; § ERNAM-Nanotechnology Research and Application Center, Erciyes University, Kayseri 38039, Turkey; ∥ ERKAM-Clinical-Engineering Research and Implementation Center, Erciyes University, Kayseri 38030, Turkey

## Abstract

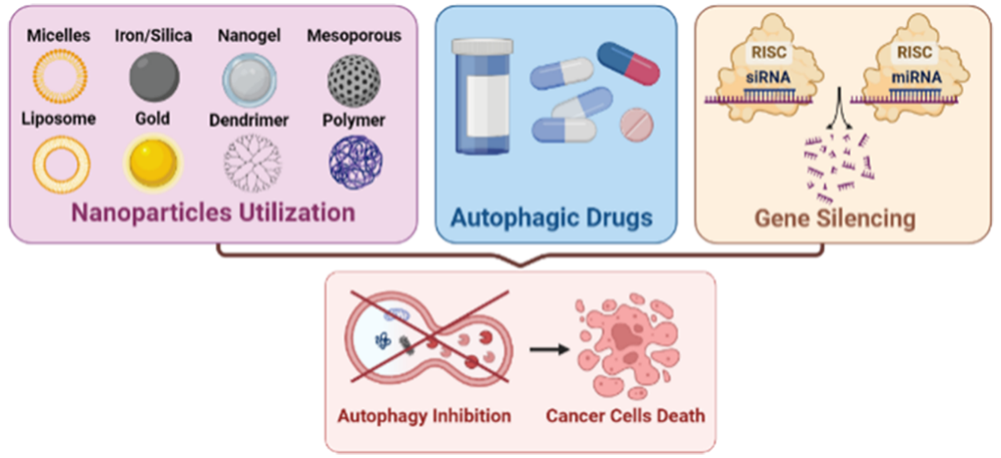

The complicated stepwise lysosomal degradation process known as autophagy is in charge of destroying and eliminating damaged organelles and defective cytoplasmic components. This mechanism promotes metabolic adaptability and nutrition recycling. Autophagy functions as a quality control mechanism in cells that support homeostasis and redox balance under normal circumstances. However, the role of autophagy in cancer is controversial because, mostly depending on the stage of the tumor, it may either suppress or support the disease. While autophagy delays the onset of tumors and slows the dissemination of cancer in the early stages of tumorigenesis, numerous studies demonstrate that autophagy promotes the development and spread of tumors as well as the evolution and development of resistance to several anticancer drugs in advanced cancer stages. In this Review, we primarily emphasize the therapeutic role of autophagy inhibition in improving the treatment of multiple cancers and give a broad overview of how its inhibition modulates cancer responses. There have been various attempts to inhibit autophagy, including the use of autophagy inhibitor drugs, gene silencing therapy (RNA interference), and nanoparticles. In this Review, all these topics are thoroughly covered and illustrated by recent studies and field investigations.

## 1 Introduction

### 1.1 What Is Autophagy?

**Figure 1:**
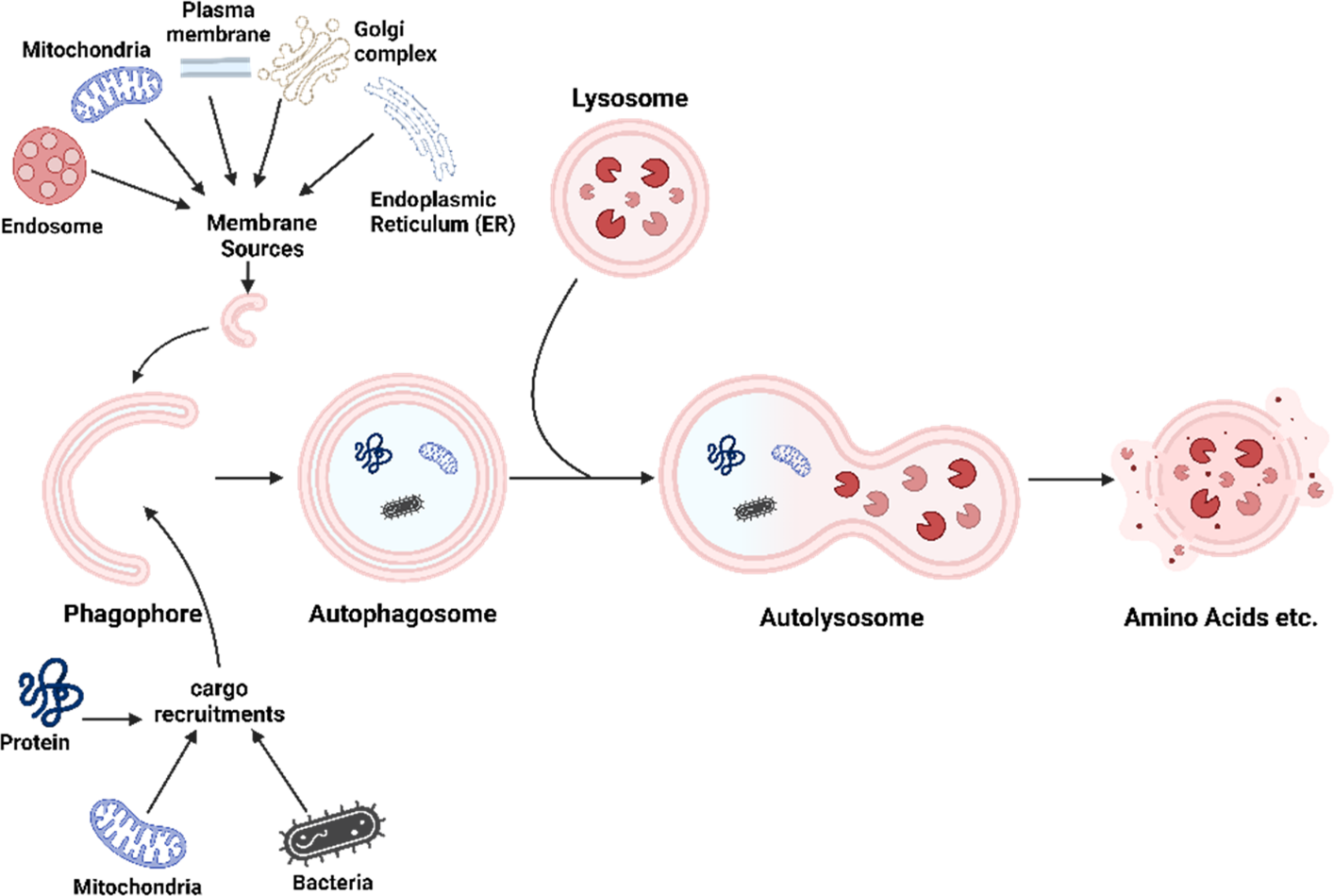
Macroautophagy process overview. The figure illustrates the macroautophagy pathway, showcasing the sequential steps from the formation of phagophores, derived from organelles like the Golgi complex, endosomes, ER, mitochondria, and plasma membrane, to their maturation into autophagosomes enclosing cellular cargo, followed by fusion with lysosomes to form autolysosomes, where cargo undergoes degradation by lysosomal enzymes, facilitating cellular recycling and maintenance.

Autophagy is a complex stepwise lysosomal degradation process that is responsible for degrading and removing dysfunctional cytoplasmic materials and damaged organelles, which supports nutrient recycling and metabolic adaptation. Autophagy represents a solid tool that cells employ in order to escape stress, being known as a process that regulates cancer.^1^ There are three different kinds of autophagy classified: microautophagy, chaperone-mediated autophagy and macroautophagy, with all focus being centered on the macroautophagy, which would be referred as autophagy throughout the context.^2^ The difference between these subtypes of autophagy mainly depends on the way the cargos are transferred into lysosomes.^3^ In microautophagy, cargos are simply engulfed and internalized directly into lysosomes as a consequence of lysosomal membrane invaginations,^4^ whereas chaperone-mediated autophagy requires a specific recognition of the cargos (proteins) that are going to be delivered to lysosomes, usually being marked with the amino acid motif (KFERQ), by another set of heat shock proteins (HSC70) for the degradation to be processed.^5^ Macroautophagy, the main common pathway of autophagy, is characterized by the formation of multiple membrane structures, starting with phagophores formation that is derived mainly from Golgi complex, endosomes, the endoplasmic reticulum (ER), mitochondria, and the plasma membrane.^6^ Phagophores mature into autophagosomes which thoroughly enclose the cargo within and then fuse with lysosomes to form structures called autolysosomes. Inside the autolysosomes, cargos got degraded completely by lysosomal enzymes^5^ (Figure 1).

Autophagy represents an intricate pathway controlled by the expression of almost 20 autophagy-related genes (ATGs),^7^ consisting of five distinguished phases; initiation, elongation, closure, maturation, and degradation.^8^ Among the ATGs, which have been first specified in yeast *Saccharomyces* cerevisiae some time ago,^9^ ATG5, ATG7, ATG12, and the major markers of autophagic activity; Beclin-1, microtubule associated light chain B (LC3B), one of the LC3 isoforms, and p62/SQSTM1 (hereafter p62) are by far the most investigated and associated with various inflammatory disorders^10,11^ as well as cancer.^12−14^ LC3 protein plays a crucial function in mammalian autophagy^7^ since it enables cargo receipt into autophagosomes and has the distinction of being the only protein to exist on these structures, making it a unique marker of autophagosome formation.^15^ There are two forms of LC3, the cytosolic LC3-I and the membrane-conjugated form, LC3-II.^16^ Under varied stresses, a modification of LC3-I to LC3-II by conjugating LC3-I with phosphatidylethanolamine (PE) is being stimulated, which leads LC3-II to bind to the membrane of the autophagosome.^17^ Becline-1, a Bcl-2-homology (BH)-3-only protein found largely in cytoplasmic structures, regulates autophagosome production (size and number) through being involved in class III phosphoinositol 3 phosphate kinase (PI3K) complex.^18^ Beclin-1, considered as a necessary component for the initiation of autophagy, takes part in the very early stage of autophagosome formation (nucleation phase).^19^ p62 is an autophagy adapter protein that interacts with ubiquitinated cargo and LC3B to carry out autophagic degradation. In this process, p62 is preferentially taken up by autophagosomes and digested by autolysosomes. As a result, decreased levels of p62 are linked to an active autophagy pathway.^20^

Autophagy is a housekeeping process. Under normal conditions, autophagy serves as a quality control mechanism in cells, helping to maintain homeostasis and redox balance by digesting nonfunctional proteins and damaged organelles, collaborating with the adaptive immune system,^21,22^ and enabling survival during malnutrition situations by recycling cellular elements to regenerate energy.^8^ However, in cases of autophagy failure in immunologic ailments and neurodegenerative illnesses such as Alzheimer’s and Parkinson’s disease, aberrant, malfunctioning proteins and organelles accumulate, leading to the development of disease.^23,24^ However, in abnormal conditions like cancer, its role is complex, acting as a double-edged sword, as will be illustrated in the next section.^25,26^

### 1.2 Role of Autophagy in Cancer

Autophagy is a process that helps cells survive under conditions of metabolic stress, such as nutrient starvation or a hypoxic environment. However, it can also play a role in cell death. The specific way in which autophagy causes cell death is not yet clear; however, when there is high metabolic stress, excessively activated autophagy can result in a type of programmed cell death called autophagic cell death.^27,28^ Recent studies on the relationship between autophagy and cancer have revealed that autophagy plays a significant role in regulating cancer development and in the response of tumor cells to cancer treatment. However, autophagy can become disrupted or overactivated in cancer cells, which makes it difficult to understand the effects it has on cancer progression and development.

Recent studies have shed light on the intricate relationship between autophagy and cancer, unveiling its profound influence on cancer development and response to therapy. However, the role of autophagy in cancer is far from static, marked by dynamic fluctuations that stem from disruptions or overactivation of autophagic processes within cancer cells.

This dynamic interplay complicates our understanding of autophagy’s impact on cancer progression, with its effects varying depending on factors such as the type and stage of the cancer, the unique genetic landscape of the tumor,^29^ and the level of autophagy activation,^30^ with the tumor stage being a primary determinant.^25,26^ A moderate level of autophagy protects the tumor from an unfavorable external environment and promotes its growth. On the other hand, excessive autophagy levels trigger autophagic death of tumor cells, and many researchers have utilized this to induce apoptosis in tumor cells and achieved remarkable results.^31−33^

Focusing on the consideration of the tumor stage, in the earliest stages of tumorigenesis, autophagy acts as a tumor inhibitor by maintaining homeostasis through the clearance of old, nonfunctional proteins and deteriorated organelles,^30^ as well as by inhibiting malignant necrosis and inflammatory responses.^34^ Studies have shown that Beclin-1 is frequently deleted in breast, ovarian, and prostate malignancies, and its absence leads to a decrement in autophagy and promotes cellular proliferation.^35^ Bif-1 is another protein that regulates autophagy by interacting with Beclin-1, and its knockout inhibits autophagy, leading to an increase in cancer formation.^36^ Homozygous deletion of ATG5 causes liver cancer in a high-penetrance animal model.^37^ However, mutations in ATG5 have been discovered in 135 patient samples with gastric cancer, colorectal cancer, and hepatocellular carcinoma.^38^

**Figure 2:**
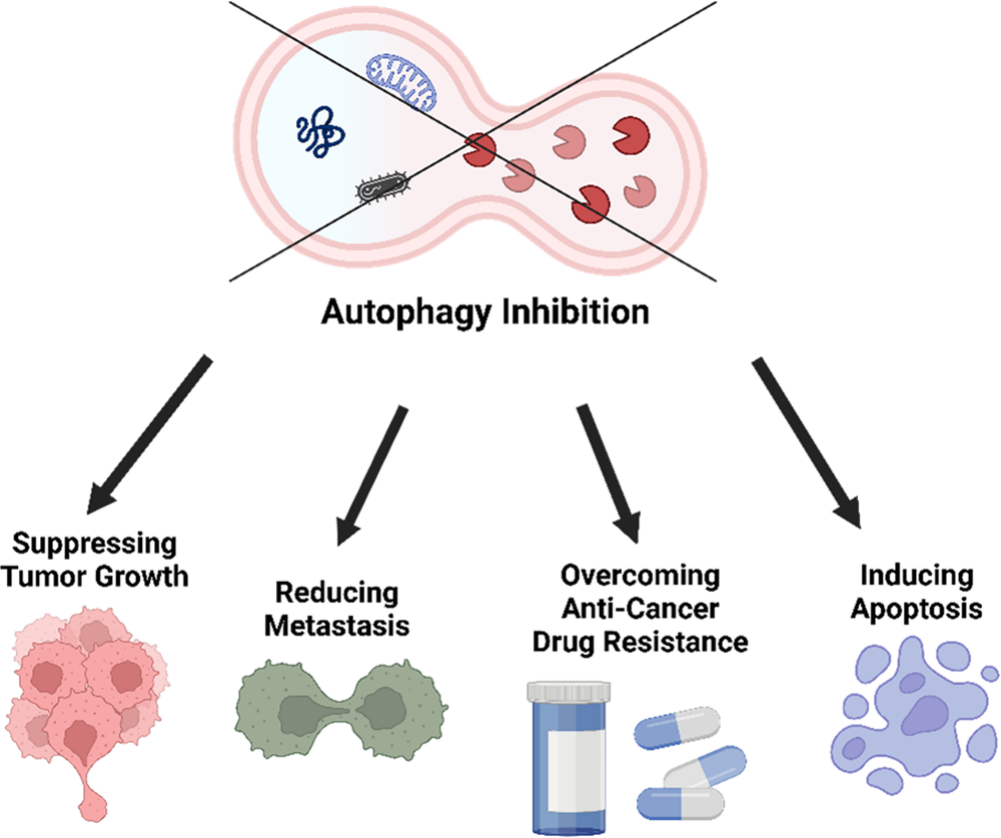
Summary of the key impacts of autophagy inhibition on cancer cells, as represented by tumor growth suppression, tumor metastasis reduction, overcoming anticancer treatment resistance, and apoptosis activation.

However, in established cancers, a plurality of data sources suggest the ability of autophagy to promote tumor growth, survival, and colonization by reducing DNA damage, maintaining functional mitochondria, dealing with cellular stresses including hypoxia, nutritional deficiency, and anticancer drug treatments,^39−42^ and meeting the high metabolic demands of the tumor in terms of nutritional supply.^43,44^ The rapid proliferation of tumor cells creates a high demand for nutrients. In the limited nutrient environment of the tumor microenvironment, autophagy can promote interaction between the tumor and the matrix, thereby fostering tumor growth.^30^ A recent investigation reveals that blocking mitophagy, the selective autophagy of mitochondria, could serve as a tactic to bolster the efficacy of cancer therapies. This research demonstrates that when an anticancer medication is paired with liensinine, a substance known to impede mitophagy, the combined treatment yields synergistic effects in eradicating cancerous cells.^45^ Altogether, while autophagy seems to be critically important in protecting normal cells from tumorigenesis initiation, inhibiting it is favorable when dealing with established and developed cancers^46−48^ (Figure 2). This could aid in blocking autophagy in cancer cells, leading to a decrease in tumor growth, less spread of tumors, overcoming resistance to cancer treatments, and activating apoptosis.

The following sections will delve deeper into the specific mechanisms in which autophagy inhibition enhances tumor therapy and will explore various approaches for inhibiting autophagy in cancer treatment.

Multiple sensors and key regulators, including proto-oncogenes (PI3K, AKT, mammalian Target of Rapamycin (mTOR), RAS and RAF), tumor suppressors (Beclin-1, TP53, FOXO1 and BCL-2), and microRNAs (miRNAs), have been identified in the analysis of autophagy inhibition or induction.^49,50^

Autophagy suppression has been found to cause tumor cell death,^51^ such as in breast cancer.^52^ As an example, in a polyoma middle T-driven model of breast cancer, a considerable delay in both tumor initiation and progression has been noticed by knocking down the Fip200 autophagy gene.^53^ In a set of studies, mouse models with deleted ATG5 or ATG7 autophagy genes have been established, in which a decrement/prevention in tumor growth has been observed.^54−56^ Using immunohistochemical analysis, it was revealed that autophagy-related genes are highly expressed in triple-negative breast cancer (TNBC) clinical samples.^57^ It has also been shown that downregulation of the LC3 gene significantly reduced cell viability and blocked the main cell mechanisms: proliferation, invasion, migration, and resistance to apoptosis in TNBC.^58^ Tumor growth in pancreatic ductal adenocarcinoma (PDAC) was slowed in one study by suppressing autophagy with autophagy inhibitors and gene silencing tools.^50^ Another finding indicated that inhibition of autophagy leads to decreased tumor growth in PDAC and suggested that autophagy inhibition could be an effective therapeutic strategy for PDAC, independent of the genetic background of the tumor cells.^59^

Several studies have shown that autophagy contributes to inflammation in the tumor microenvironment and adjacent cells, promoting tumorigenesis. Toll-like receptors (TLRs), known modulators of inflammatory responses, can influence autophagy activity, impacting tumor progression. Studies have shown that TLR4 activation and TLR9 upregulation can induce autophagy and contribute to cell migration, invasion, and proliferation in cancer.^60,61^ Similarly, the Receptor for Advanced Glycation End-products (RAGE) has been linked to both inflammation and autophagy activation. A study suggests that promoting autophagy via RAGE expression may contribute to cell survival and protection against oxidative stress-induced damage in pancreatic tumor cells.^62^

Additionally, Several ATGs, such as ATG5, ATG7, and ATG16L1, are directly involved in regulating the production of pro-inflammatory cytokines and reactive oxygen species (ROS).^63^ ROS, a byproduct of cellular metabolism, can stimulate autophagy, further influencing inflammatory responses within tumors.^64,63−67^ Studies have shown that increased ROS levels can stimulate autophagy and enhance cancer cell survival in various cancers.^68−70^ Moreover, inflammatory cytokines such as TNF-α, IL-6, and IL-8 can modulate autophagic activity in cancer cells, affecting tumor development in complex ways.^71−74^

Nevertheless, autophagy has also been correlated to tumor metastasis by promoting cancer’s aggressiveness.^75,76^ Because metastatic tumor cells must adapt to a different milieu than the initial site^77^ and overcome a number of obstacles in order to develop colonies, there is strong evidence that autophagy plays a significant role in facilitating this type of adaptation.^78,79^ Many studies have been reported indicating that high levels of LC3 and other ATGs are correlated to cancer progression, implying the critical role autophagy plays in metastasis.^80−83^ According to one study, increased expression of LC3B and ATG17 is related to a considerably lower survival time in TNBC patients.^84^ Also, upregulated expression of ATG7 in breast cancer tissue is highly related with lower overall survival and distant metastasis-free survival in patients.^85^ Nonetheless, tumoral expression of autophagy-related proteins has been found to be higher in brain metastasis than in primary breast cancers.^86^ In human breast cancer, greater punctate staining for LC3B was correlated with lymph node metastases and shorter survival.^82,83^ In hepatocellular carcinoma, LC3B expression was also associated with metastasis, with increased LC3B staining in metastases compared to primary tumors.^76,87^ Increased expression of an autophagy gene signature was linked to a more aggressive and invasive phenotype in human glioblastoma.^80^

Nevertheless, autophagy has also been recently proposed as potential cause for evolution and progression of resistance toward several anticancer agents.^88−92^ And once drug resistance takes place, cancer cells aggressiveness and metastasis became inevitable which bring about failure in the treatment and eventual motility.^93^ Several investigations have shown that inhibiting autophagy boosts the lethal effects of chemotherapeutic drugs, emphasizing the importance of autophagy in stress tolerance and drug resistance.^94−98^ For example, retroviral shRNA suppression of the ETS transcription factor ELK3, a signaling protein involved in autophagy activation, decreased autophagy and improved doxorubicin (DOX) responsiveness, one of the most frequently used drugs in treating breast cancer.^99^ In another study, miR-520b significantly improved DOX sensitivity in hepatocellular carcinoma (HCC) via suppressing ATG7-dependent autophagy.^100^ Yet, blocking autophagy, as indicated by the cell surface molecule CD24, resulted in a significant increase in sorafenib sensitivity, the first US Food and Medicine Administration-approved drug for targeted HCC treatment.^101^ It has been demonstrated that autophagy suppression increased chemo-sensitivity and promoted apoptosis in cholangiocarcinoma.^102,103^

Autophagy plays a key role in cell death decisions and has the ability to block apoptosis.^104^ The most likely causes are autophagy’s ability to activate mitophagy and diminish the amount of pro-apoptotic proteins in the cytosol.^105^ For example, when autophagy is inhibited, the pro-apoptotic protein PUMA is elevated.^106^ Anoikis, or the lack of extracellular matrix (ECM) attachment, causes cancer cells to die through apoptosis. Nevertheless, autophagy has been proven to help ECM-detached cancer cells evade anoikis and survive.^107^ It has been demonstrated that inhibiting autophagy reduces glycolytic capability, oxidative phosphorylation, and cell proliferation, while also increasing apoptosis in in vitro and in vivo studies.^108,109^ According to one study, inhibiting autophagic flux in quiescent breast cancer cells increases the cumulating of damaged mitochondria and ROS, leading to apoptosis.^110^ Another study found that utilizing the lucanthone drug, a possible sensitizer to chemotherapy and radiation, caused lysosomal dysfunction, autophagy suppression, and apoptosis in cancer cells.^111^ Nonetheless, suppressing autophagy in cancer cells increased the apoptotic effect of paclitaxel (PTX), an apoptosis inducer used to treat lung adenocarcinoma.^112^ In a recent study, inhibiting autophagy was found to sensitize apoptosis in cancer cells by FOXO3a Turnover, an autophagy-regulating transcription factor.^104^

Accordingly, great efforts have been made in order to inhibit autophagy as a mechanism for enhancing cancer treatment therapies^113^ using different methods in order to accomplish the inhibition including the use of autophagy inhibitor drugs, knockdown critical ATG genes by RNA interference (RNAi) and utilizing nanoparticles.^114,115^

## 2 Traditional Medicine for Autophagy

**Figure 3:**
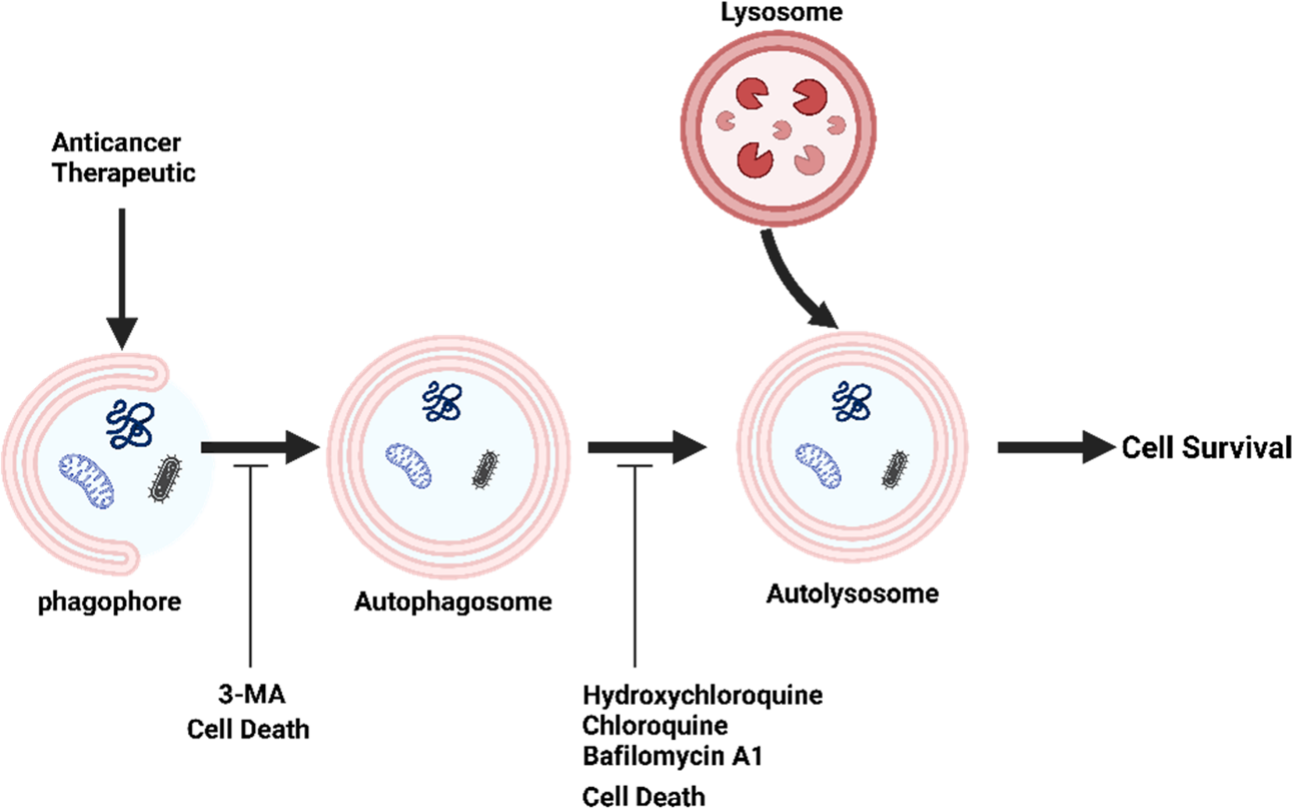
The most common autophagy inhibitors work to inhibit autophagy. 3-MA prevents the formation of the autophagosomes, whereas CQ, HCQ and BafA1 work by inhibiting autophagosome–lysosome fusion, leading to accumulation of autophagosomes.

Nowadays, there are a variety of drug agents that block autophagy at its various stages, ranging from autophagy induction and autophagosome formation through lysosomal degradation,^116^ headed by chloroquine (CQ), hydroxychloroquine (HCQ), 3- methyladenine (3-MA) and Bafilomycin A1 (BafA1), with CQ and HCQ being the most investigated in clinics^117^ (Figure 3). Several studies have shown that combining these autophagy inhibitors with front-line cancer medicines enhances their anticancer capabilities and promotes cell death.^12,118−121^

CQ and HCQ are the most commonly used medications for chronic inflammatory disorders such rheumatoid arthritis,^122^ systemic lupus erythematosus,^123^ and sarcoidosis,^124^ and have been used to limit the spread of melanoma, pancreatic cancer, and bladder cancer by blocking autophagy.^116,125^ They have also been used to make cancer cells in chemo-resistant and radio-resistant tumors more susceptible to various treatments.^119,126^

CQ was developed in 1934 and has primarily been used to treat malaria.^127^ It has been recently employed as an autophagy inhibitor since it has been shown to impede the functionality of lysosomes by causing a stoppage in the fusion of autophagosomes and lysosomes.^128^ HCQ, a CQ derivative, suppresses autophagy by manipulating the pH of lysosomes, which prevents autophagosome destruction.^129,130^ Because HCQ is less hazardous than CQ at peak doses, it has been commonly used in clinical trials over CQ.^129,131^ CQ has been utilized in glioma cells to see whether there are any synergistic effects that may be produced by mixing Temozolomide, a medicine used to treat brain malignancies, with autophagy inhibitory agents, and the results reveal that inhibiting autophagy with CQ can help to prevent cancer growth.^118^ Furthermore, by inhibiting autophagy, CQ suppressed tumor growth in PDAC mice models.^56^ HCQ inhibited autophagy in cancer stem-like cells, which influenced HCC cell invasion and migration, as well as EMT, a critical stage in the progression of cancer metastasis.^132^ The combination of Thymoquinone, a major bioactive ingredient isolated from black cumin,^133^ and CQ triggered apoptosis in cancer cells in glioblastoma.^134^ In one study, treatment with CQ coupled with artemisinin, an element derived from sweet wormwood,^135^ induced lung cancer cells to apoptosis in a synergistic manner.^136^ Combining the effective autophagy inhibitor, mTOR inhibitor, with HCQ increased six-month median progression-free survival (PFS) in breast cancer patients, according to a phase II trial.^137^ CQ treatment caused apoptosis in HeLa cells, which was determined by DNA breakage.^138^ However, combining CQ with cisplatin, a chemotherapy treatment, activated apoptosis by suppressing autophagy, as seen by an accumulation of ubiquitinated misfolded intracellular proteins such as Beclin-1 and LC3-II, which increased the drug’s efficacy.^12^ CQ boosted the anticancer effects of 5-fluorouracil (5-FU), cytotoxic chemotherapeutic drug, on human colorectal cancer cells while inhibiting colony forming abilities, as pretreatment with CQ caused cell cycle arrest at phase G0/G1.^119^ Furthermore, using CQ in combination with a class of cancer drugs such sunitinib, bevacizumab, and oxaliplatin (OX) boosted apoptosis and sensitivity in colon cancer cells under hypoxic circumstances.^120,121^ In a recent study, HCC cells were treated with a combined treatment of HCQ and sorafenib, which reduced tumor growth, cellular proliferation, migration, and invasiveness by suppressing autophagy and regulating apoptosis, overcoming drug resistance.^139^ In a recent study, a nanovesicle known as DC-DIV/C was designed to deliver CQ and DOX-HCl simultaneously to cancer cells that were resistant to the drug. Both in vitro and in vivo experiments showed that this codelivery suppressed autophagy and hence enhanced the antitumor effects of the treatment.^140^

3-MA is an autophagy inhibitor that inhibits the development of autophagosomes by altering PI3K, a critical autophagy’s arranger.^141^ It is been proven that suppressing autophagy with 3-MA improves chemotherapeutic medication efficacy in laryngeal cancer.^142,143^ In a recent study, treating HCC cells, HepG2, with sorafenib and 3-MA at the same time inhibited cell proliferation while also triggering apoptosis. Thus, by suppressing autophagy, MA may be able to reduce drug resistance and improve drug efficiency.^144^ In the same manner, in colon cancer, OX resistance has been overcome by blocking autophagy. CT26 cells were cotreated with OX and 3-MA, which increased apoptosis and inhibited tumor growth in vivo, as well as extending animal survival time.^145^ 3-MA enhanced Tocomin apoptosis, an available commercial drug with antitumor characteristics made up of vitamin E components, in breast cancer cells.^146^ Autophagy provides tumor cells with an adaptability to survive under starving situations, as previously indicated. In a recent study, dendritic mesoporous organosilica nanoparticles (DMONs) loaded with glucose oxidase, a starvation inducer, and 3-MA have been created as a method for improving cancer starvation therapies. Autophagy inhibition has been shown to improve the efficacy of starvation treatment, resulting in cancer progression control.^147^ In another study, a nanocarrier system has been developed based on the use of functionalized mesoporous silica nanoparticles and Temozolomide, in which its anticancer activity was further increased when combined with 3-MA.^148^

BafA1, a Streptomyces gresius-derived antibiotic, suppresses autophagy by blocking the acidification of endosomes and lysosomes, which prevents their union.^149^ By suppressing autophagy in colorectal cancer cells, BafA1 may be able to prevent cell cycle transition and increase cell death.^150,151^ BafA1 also stopped prostate cancer cells from spreading by blocking autophagy.^152^ BafA1 suppressed the growth of TNBC cells and caused an increase in the levels of p62 and the ratio of LC3-II to LC3-I.^153^. In tongue squamous cell carcinoma (TSCC), BafA1 suppressed autophagy and increased the susceptibility of the cells to cisplatin.^154^ The same effect was shown in small cell lung carcinoma, where the use of BafA1 increased cisplatin cytotoxicity.^155^ In HCC, BafA1 treatment inhibited cellular proliferation, induced cell cycle arrest, prompted Cyclin D1 turnover, and caused caspase-independent apoptosis in both 2-dimensional and 3-dimensional cultures. All of this was caused by autophagy suppression, which was demonstrated by an increase in LC3 conversion.^156^ In breast cancer, epirubicin cytotoxicity, a commonly used medication to treat breast cancer, was elevated when MDA-MB-231 and SK-BR-3 cell lines were treated with bafilomycin A1.^157^ Furthermore, combining RAD001, an mTOR inhibitor, with BafA1 increased mTOR cytotoxicity, decreased cell viability, and promoted apoptosis in bladder cancer cells.^158^ Moreover, treatment with BafA1 improved the cytotoxicity of cytarabine, a chemotherapeutic medication used to treat leukemia in acute myeloid leukemia (AML) cells.^159^

Studies on autophagic medicines are summarized in Table 1.

**Table 1:** Summary of the Studies on Autophagic Medicines

Tumor Type	Autophagy Inhibitor	Additional Drug/Agent	Results	Ref
Brain malignancy/glioma	CQ	Temozolomide	Preventing cancer growth	(118)
Hepatocellular carcinoma	HCQ	–	Overcoming cell invasion and migration	(132)
Glioblastoma	CQ	Thymoquinone	Triggering apoptosis	(134)
Lung cancer	CQ	Artemisinin	Enhancing apoptosis in a synergistic manner	(136)
Breast cancer	HCQ	mTOR inhibitor	Increasing six-month median PFS	(137)
Cervical cancer	CQ	–	Causing apoptosis	(138)
Human cervical cancer	CQ	Cisplatin	Activating apoptosis and boosting cisplatin’s efficacy	(12)
Colorectal cancer	CQ	5-Fluorouracil (5-FU)	Inhibiting colony forming abilities	(119)
Breast, cervical, colorectal, hepatocellular, laryngeal and prostate cancer cell lines	CQ	Sunitinib	Enhancing apoptosis and cell’s drug sensitivity	(120)
Colon cancer	CQ	Bevacizumab and Oxaliplatin	Enhancing sensitivity to oxaliplatin under normal and hypoxic conditions in a synergistic manner	(121)
Hepatocellular carcinoma	HCQ	Sorafenib	Reducing tumor growth, cellular proliferation, migration, and invasiveness	(139)
Chronic myeloid leukemia cancer and breast cancer	CQ	DC-DIV/C and DOX-HCl	Increasing antitumor efficacy (with 84.52% tumor inhibition rate on K562/ADR tumor)	(140)
Hepatocellular carcinoma	3-MA	Sorafenib	Inhibiting cell proliferation and triggering apoptosis	(144)
Colon cancer	3-MA	Oxaliplatin	Increasing apoptosis and Inhibiting tumor growth in vivo	(145)
Breast cancer	3-MA	Tocomin	Enhancing apoptosis	(146)
Breast cancer	3-MA	DMONs loaded with glucose oxidase	Improving the efficacy of starvation treatment	(147)
Glioma	3-MA	mesoporous silica nanoparticles- Temozolomide	Enhancing anticancer activity	(148)
Prostate cancer	BafA1	–	Preventing the spread of cancer cells	(152)
Tongue squamous cell carcinoma (TSCC)	BafA1	Cisplatin	Increasing the susceptibility of the cells to cisplatin	(154)
Small cell lung carcinoma	BafA1	Cisplatin	Increasing cisplatin cytotoxicity	(155)
Hepatocellular carcinoma	BafA1	–	Inhibiting cellular proliferation, inducing cell cycle arrest, and causing caspase-independent apoptosis	(156)
Breast cancer	BafA1	Epirubicin	Elevating epirubicin cytotoxicity	(157)
Bladder cancer	BafA1	RAD001, an mTOR inhibitor	Increasing mTOR cytotoxicity, decreasing cell viability, and promoting apoptosis	(158)
Leukemia in acute myeloid leukemia (AML) cells	BafA1	Cytarabine	Improving the cytotoxicity of cytarabine	(159)

## 3 Developed and Promising Therapy for Inhibition of Autophagy

### 3.1 Gene Silencing Therapy

RNAi, which was first reported in 2006 and involves the use of microRNAs (miRNAs) or small interfering RNAs (siRNAs) to inhibit the expression of certain genes, is swiftly gaining traction as a powerful technique for gene silencing.^160^ Both of them are short RNA duplexes that contain an average of 21–23 nucleotides.^161^ Despite the structural and functional similarities between miRNAs and siRNAs, there are some significant distinctions between them. The primary distinction between the two molecules is found in their modes of action.^162^ While both act by promoting the inhibition of messenger mRNA (mRNA) expression, siRNAs work by cleaving mRNAs, while miRNAs work by blocking mRNA translation into proposed proteins. In addition to their modes of action, they differ in the way they recognize target mRNA. To have an effect, siRNA must be completely complementary to its target mRNA, allowing it to be used to silence a single target gene. For miRNA, however, a partial interaction between miRNA and its target is sufficient to cause it to act. As a result, it might be utilized to block several genes.^162^

Nowadays, these technologies are employed to inhibit autophagy by focusing on key ATG genes implicated in the process as a successful method to boost the antitumor response while lowering cancer progression.^1,163^

#### 3.1.1 miRNA Utilization

miRNAs have a critical role in cancer formation, growth, and metastasis regulation,^164,165^ being found that about 50% of miRNA genes are located at fragile sites in the genome or in cancer-related regions.^166^ As a result, miRNA-mediated RNAi has emerged as a promising technique for killing cancer cells by controlling a variety of cell activities such as apoptosis, autophagy, proliferation, differentiation, invasion, metastasis, and stress.^167−172^ Many research have promoted the idea of delivering miRNAs that might silence certain autophagic genes, potentially overcoming anticancer drug resistance.^173−175^

MicroRNA-30a (miR-30a) has been shown to regulate rapamycin-induced autophagy in lung and breast cancer cells^176^ while also inhibiting autophagy in cancer cells via targeting Beclin-1.^176,177^ For example, miR-30a mimics boosted the sensitivity of imatinib, a medication that inhibits the BCR-ABL tyrosine kinase responsible for chronic myeloid leukemia (CML) in a targeted manner, and promoted mitochondria-dependent intrinsic apoptosis in vitro and in vivo.^115^ Similarly, by suppressing autophagy, miR-30a enhanced imatinib sensitivity and elevated apoptosis in CML cells by downregulating BECN1 and ATG5 levels.^178^ Furthermore, delivering miR-30a improved cisplatin’s ability to induce cell apoptosis by silencing Beclin-1 gene,^177^ where comparable findings were obtained by modulating miR-885–3p.^179^ In a recent study, it was discovered that blocking autophagy using miR-30a enhanced cisplatin efficiency in ovarian cancer by regulating the activation of the TGF-β/Smad4 pathway.^180^ In another study, miR-30a was found to improve nonsmall-cell lung cancer (NSCLC) outcome after neoadjuvant chemotherapy via suppressing autophagy and accelerating NSCLC cell death.^181^ Nonetheless, in another study it has been discovered that miR-30a elevates prostate cancer radiosensitivity by suppressing autophagy.^182^ miR-30a-5p could also slow the growth of lung squamous cell carcinoma by downregulating ATG5 and thereby blocking autophagy in vitro and in vivo.^183^

MicroRNA-101 (miR-101) represents a key regulator that is downregulated in a variety of malignancies, including liver, prostate, breast, bladder, colon, and pancreatic cancers.^184−186^ miR-101 inhibits the Zeste homologue 2 (EZH2) and myeloid cell leukemia-1 (Mcl-1) genes, causing apoptosis to increase, proliferation to decrease, and metastatic tumor growth to be prevented.^184,186^ Furthermore, miR-101 suppresses the RAB5A, ATG4C, and ATG4D genes, making it a primary autophagy mediator.^187^ For example, miR-101 boosted the chemo-sensitivity of tamoxifen in breast cancer cells by inhibiting the autophagic pathway, which improved treatment outcomes.^188^ HCC cells have been shown to be more sensitized to doxorubicin and fluorouracil therapy when autophagy is inhibited by miR-101.^189^ Another study found that inhibiting autophagy via miR-101 can improve cisplatin cytotoxicity in HCC cells and hence increase apoptosis population.^190^

UNC51-like kinase 1 (ULK1) is one of the ATGs that participates in the formation of autophagosomes,^191^ and when it is inhibited, cancer cells exhibit an effective reduction of autophagy and an increase in apoptosis.^192,193^ For instance, a reduction in the proliferation, migration, invasion, and autophagy of pancreatic cancer has been seen by inhibiting ULK1 via miR-372.^194^ Recent research found that miR-373 inhibited ULK1, which in turn promoted apoptosis in cholangiocarcinoma cells via suppressing autophagy. LC3-II/LC3-I value and Beclin-1 protein level were down regulated while p62 protein was markedly raised, indicating an inhibition of autophagy.

Numerous other research in this area have taken use of various miRNAs that inhibit the expression of genes associated with autophagy, hence decreasing autophagy and improving the effectiveness of treatment. In one study, it was demonstrated that by reducing autophagy activity by suppressing ATG12 through miR-23b, radiotherapy effectiveness was improved in pancreatic cancer cells.^13^ Additionally, miR-29c inhibited autophagy and elevated gemcitabine-induced apoptosis, a cytotoxic drug used to treat some cancers, in pancreatic cancer by regulating the expression of USP22.^195^ Similar to this, miR-29a decreased autophagic activity and made pancreatic cancer cells more susceptible to gemcitabine therapy.^196^ Using miR-410-3p to target high mobility group box 1 (HMGB1), a critical autophagy regulator that influences inflammation, tumor cell motility, and metastasis,^197,198^ enhanced chemo-sensitivity by preventing cancer cells from inducing autophagy.^199^ In another study, targeting HMGB1 specifically by miR-34a enhanced cell death and blocked autophagy in AML.^200^ By targeting ATG7, miR-375 prevented the conversion of LC3-I to LC3-II in HCC cells, which reduced autophagy and the proliferation of cancer cells.^201^ Another study found that miR-590-5p targets the autophagy protein ATG3 and blocks autophagy, increasing the radiosensitivity of PDAC cells.^202^

In a recent study, miR-373-3p suppressed autophagy by targeting AKT1 in cervical cancer, which limited the growth of the tumor both in vitro and in vivo.^203^ Furthermore, a recent study found that miR-338 might target ATF2 via the mTOR signaling pathway to suppress the proliferation and autophagy of cervical cancer cells, indicating the possible use of miR-338 in the treatment of this disease.^204^ In a another study, miR-373 was used to target the autophagy-upregulated proteins CD44 and TGFBR2 in glioblastoma multiforme (GBM), which prevented the cancer cells’ migration and invasion.^205^

Numerous other miRNAs, such as miR-216a, miR-30d, miR-205, miR-199a-5p, and miR-885-3p,^177,179,206−208^ have been used to block autophagy and make cancer cells more sensitive to radiation or chemotherapy. Therefore, it is evident that miRNAs may offer a novel treatment strategy for improving the prognosis of cancer patients by blocking autophagy.

#### 3.1.2 siRNA Utilization

siRNA is a potent tool that has been used widely in cancer treatment in both preclinical (in vitro and in vivo) and clinical studies. It has been approved for use in treating adults with hATTR amyloidosis^209^ and acute hepatic porphyria^210^ under the brand names ONPATTRO (Patisiran) and GIVLAARITM (Givosiran). Delivering siRNAs that specifically target autophagic genes, primarily ATG5 and ATG7, has been advocated in numerous studies as a way to potentially overcome anticancer medication resistance.

In one study, six of the main ATGs, Beclin-1, ATG-3, ATG-4b, ATG-4c, ATG-5, and ATG-12, have been successfully suppressed utilizing particular target-siRNAs. By employing siRNAs to block these genes, autophagy was suppressed, as was seen by a reduced concentration of autophagosomes. Consequently, radiation-resistant cancer cells become increasingly susceptible to it.^211^ In one study, using siRNA to reduce Beclin-1 increased PARP breakage, mitochondrial membrane depolarization, and cytosolic cytochrome c levels following doxorubicin therapy. Each and every one of them highlighted the considerable increase in cell apoptosis.^212^

Another study found that suppressing autophagy by utilizing siRNAs against ATG5 and ATG7 blocked autophagy that is being activated by the anticancer drug anthracycline daunorubicin (DNR). This inhibition increased the effectiveness of DNR in treating myeloid leukemia.^213^ Additionally, it has been demonstrated that the use of sorafenib resulted in the activation of autophagy as proven by an accumulation of autophagosomes in various HCC cell lines. Inhibiting the drug-related autophagy, as a result, made cancer cells more sensitive to it.^214^ Similar results were seen when Beclin-1 or ATG-5 siRNA were used to block autophagy in renal cell carcinoma (RCC) cells, increasing the sensitivity of cancer cells to sorafenib.^215^ Likewise, it has been discovered that linifanib, an effective drug with significant anticancer actions in a variety of solid tumors,^216^ induces a high amount of autophagy in HCC cells. In which the apoptosis generated by linifanib was enhanced and its effectiveness was therefore boosted by inhibiting this agent-associated autophagy with ATG5 and ATG7-siRNAs.^217^

In one study, reducing autophagy with siRNAs that targeted Beclin-1 or ATG-5 improved cancer immunotherapy as shown by a recovery in the sensitivity of hypoxic tumor cells to Cytolytic T lymphocytes (CTL)-mediated lysis. While in vivo results indicated that blocking autophagy increased cellular apoptosis and slowed cancer progression.^218^ Furthermore, S100A8 gene silencing boosted cancer cell apoptosis and the sensitivity to arsenic trioxide (As2O3), a strong environmental cocarcinogen for various human malignancies. As it was found that S100A8 knockdown caused autophagy suppression, which was made clear by a decrease in LC3-II protein levels.^219^

In addition, it has been demonstrated that ATG7 siRNA can be used to overcome PTX resistance in HeLa cells by suppressing autophagy.^220^ A demethylated derivative of cantharidin called norcantharidin (NCTD) shows anticancer properties in a number of malignancies. In one study, it was investigated whether combining autophagy inhibition with NCTD would boost the medication’s effectiveness in HepG2 cells. Based on this, autophagy inhibition was carried out using ATG5 siRNA and was verified by a reduction in the expression of the LC3-II protein. Cell apoptosis increased as a result of autophagy suppression, as shown by an increase in the expression of Bax, cytochrome c, cleaved caspase-3, caspase-9, and PARP.^221^ The same results were attained when human cholangiocarcinoma cells were treated with NCTD and autophagy was prevented by siRNA-mediated downregulation of ATG-5.^222^

Another study demonstrated that boosting apoptosis by suppressing autophagy with ATG7 siRNA made cells more sensitive to Temozolomide and curcumin either alone or in combination.^223^ In one study, epirubicin, which has been shown to promote autophagy, was combined with gene therapy using ATG-7 and ATG-5 siRNA to treat TNBC cells. Inhibiting autophagy has been demonstrated to significantly reduce the viability of cancer cells and increase apoptosis.^88^ Similar findings were found whereby blocking autophagy with ATG-7 siRNA caused epirubicin’s cytotoxicity to rise in a number of breast cancer cells, along with an increase in caspase-9- and caspase-3-dependent apoptosis.^157^

In a recent study, downregulation of LC3 gene by delivering siLC3 significantly reduced cell viability and blocked the main cell mechanisms; proliferation, invasion, migration, and resistance to apoptosis in the metastatic MDA-MB-231 cell line, which represents a simulation of TNBC.^58^ A naturally occurring kava chalcone called flavokawain B (FKB) has a potent anticancer effect on a number of cancer types that also activate autophagy. Blocking ATG5 or ATG7 expression using siRNAs prevented FKB from inducing autophagy in GBM cells, which led to the cells’ transition from senescence to death.^224^ Another recent study demonstrated that the agent Enzalutamide (ENZ), currently being studied to treat bladder cancer, boosted apoptosis when ATG5 was knocked down by siRNA.^225^ In the PTX-resistant NCI-H23, an NSCLC cell line, knocking down Beclin-1 by siRNA simultaneously decreased multidrug resistance protein 7 (ABCC10) and P-glycoprotein (P-gp), which functions as a drug efflux pump, and restored the sensitivity to PTX in vitro. In vivo, decreasing autophagy caused tumor sizes to decrease.^226^ Another study discovered that lung adenocarcinoma cells were more susceptible to the effects of Vismodegib, the first inhibitor of Sonic Hedgehog,^227^ a crucial target in cancer therapy, when autophagy was inhibited by ATG5 or ATG7-siRNAs.^228^ In recent studies, siRNA-mediated LC3 knockdown dramatically increased the efficacy of GBM treatments.^229,400^ Another study found that inhibiting autophagy by using siRNA to silence the ATG-7 gene increased the effectiveness of cisplatin as it was associated with increased apoptosis, decreased cell survival rate, decreased measurements of cell density, altered morphology of the cells, and decreased measurements of mitochondrial membrane potential in ovarian cancer SKOV3 cells.^230^

### 3.2 Nanoparticles

Nanoparticles (NPs) are particles with dimensions on the nanometer scale, typically between 1 and 100 nm.^231^ They can be made of a wide variety of materials, including metals, ceramics, polymers, and lipids, and can have unique properties due to their small size and high surface area-to-volume ratio. Recently, nanoparticles are widely used as delivery vehicles for genetic material such as DNA and RNA.^401^ These particles can protect the genetic material from degradation, increase its stability, and target it to specific cells or tissue. The nanoparticles are engineered to have certain properties such as size, surface charge, and surface chemistry that allow them to interact with the cell membrane and deliver the genetic material into the cell. Nanoparticles can also play a role in inhibiting autophagy and cause cancer cell death through various mechanisms, such as by directly targeting and disrupting the autophagic machinery within the cell,^232^ by targeting signaling pathways that regulate autophagy, such as the mTOR pathway, or by inducing oxidative stress.^233^

#### 3.2.1 Nanoparticles as Delivery Systems

**Figure 4:**
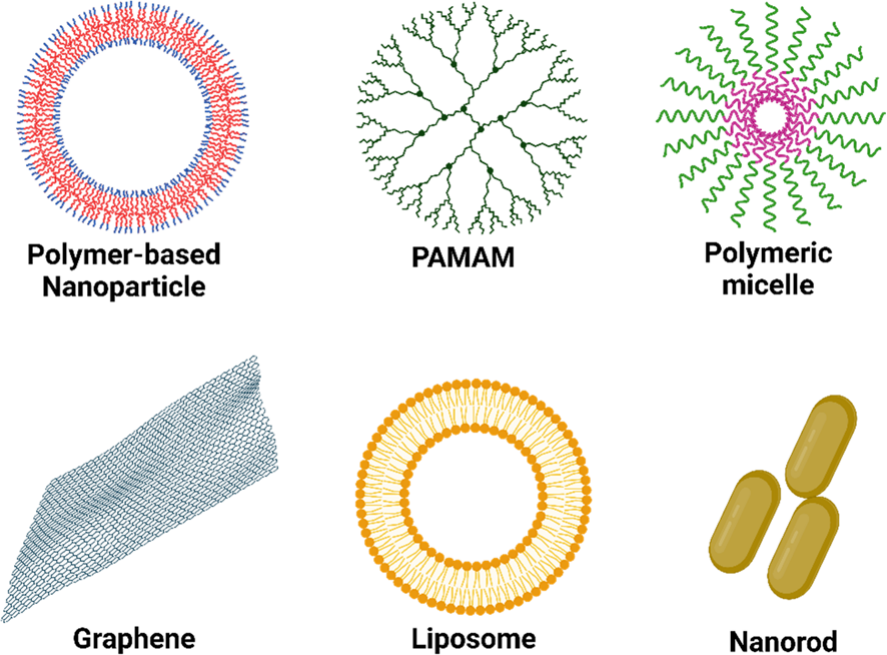
The most utilized nonviral gene vectors for transporting genetic materials: polymers, polyamidoamine dendrimers (PAMAM), micelles, graphene, liposomes, and nanorods.

Genetic therapeutic agents must be successfully introduced into cells in order to inhibit autophagy utilizing gene therapy. The quick degradation of miRNAs and siRNAs once they enter the circulation, as well as their negative charge, which prevents them from entering the cell membrane, are two of the challenges they face. Viral-based delivery systems were developed and employed for this purpose, but they were shown to be unsafe, causing significant immunological reactions as well as carcinogenic potential.^234,235^ In order to transport genetic materials and meet the many obstacles in the area, a variety of nonviral gene vectors, nanoparticles, have been used. A summary of the most commonly used vectors is shown in Figure 4.

With a median particle size in the nanometer scale, generally between 10 and 400 nm, nanocrystals made up entirely of drugs are carrier-free colloidal drug delivery vehicles.^236^ They provide a number of benefits, including high solubility, prolonged circulation, and 100% bioavailability to the target tissue or organ.^237−239^ In a recent study, a drug-delivering-drug (DDD) platform was developed, comprised of antitumor-drug nanorods, rod-like nanocrystals of PTX, which served as a vehicle for miR-101 delivery to cancer cells. In vivo, this administration resulted in an inhibition of autophagy, as evidenced by a decrease in LC3-II mRNA and an increase in p62 mRNA. The drug’s capacity to kill cancer cells was facilitated when it was combined with miR-101, as evidenced by a considerable increase in apoptotic cell death. Furthermore, when compared to administering the nanorods alone, this combination resulted in an 80% reduction in tumor volume.^240^

Polymers have been crucial to the development of drug delivery technology because they provide cyclic dosage, adjustable release of both hydrophilic and hydrophobic medicines, and controlled release of nanomedicines in constant doses over long periods of time.^241^ In one study, Beclin-1 siRNA and DOX were delivered to cancer cells simultaneously using 1,3-diol-rich hyperbranched polyglycerol (HBPO) and phenylboronic acid-tethered hyperbranched oligoethylenimine (OEI600-PBA). It has been observed that when these two hyperbranched polymers are joined together, a core–corona nanoconstruction arises by itself. This nanostructure provided enhanced siRNA affinity, additional anticancer drug loading capability, facilitated cellular transport, and acidity-responsive payload release. When siRNA and DOX were delivered together using this nanostructure, Beclin-1 gene could be successfully silenced, autophagy that is caused by DOX has been suppressed, and cell death in cultivated malignant cells was significantly increased. Comparing the in vivo combinational treatment to the DOX alone treatment, it was found to significantly increase safety while increasing the tumor’s sensitivity to DOX chemotherapy.^242^ Pullulan, a naturally occurring nonionic and linear homopolysaccharide, has gained increasing attention as a gene delivery system due to its outstanding biocompatibility, low viscosity, and preferable water solubility.^243^ One study used a pullulan-based copolymer delivery system to deliver both DOX and Beclin-1 siRNA simultaneously. Multiple components have been used in the delivery system known as FPDP in order to produce high outputs. In a study, pullulan was modified with lipophilic desoxycholic acid to produce micelles, and Beclin-1 siRNA was delivered using PEI, with folate (FA) employed for targeted delivery of the system to cancer cells. As a result, the delivery system showed excellent DOX and shBeclin1 loading capacities, practical storage stability, and a sustained drug release profile. The codelivery of DOX and Beclin-1 siRNA produced synergistic cell death, according to in vitro and in vivo results.^244^

Electrostatic interactions enable negatively charged siRNAs to be incorporated with cationic polymers such polyethylenimine (PEI), polyamidoamine dendrimer (PAMAM),^245,246^ and chitosan, which can then be formed into nanoparticles with excellent encapsulation.^247^ PAMAMs are a category of synthetic polymers which have been exploited as nanocarriers due to their distinctive qualities, including their highly branched structure, water solubility, high charge densities, and abundance of amine groups for additional functionalization.^248^ For instance, a PAMAM-based nanocarrier (PPP) has been designed in recent research in order to transfer miR-34a into gastric carcinoma cancer cells. Phenylboronic acid was used to modify the surface of PAMAM in the creation of the nanocarrier, enabling targeted distribution of the nanocarrier to cancer cells and improving endocytosis effectiveness. The results demonstrated that delivering miRNA-34a with this nanocarrier counteracted Notch-1, a signaling pathway crucial in autophagy, which resulted in inducing apoptosis and suppressing cell migration and invasion, as well as decreasing tumor growth in vivo.^245^

The “Golden Standard” nonviral gene delivery vector with respectable transfection potential and moderate immunogenicity is usually regarded as being PEI, a highly assessable synthetic polymer.^249^ In a recent study, a PEI-based delivery system was used to deliver DOX and an antiautophagy siRNA simultaneously. The resulting delivery system (PEI/Si-D) containing mirror RNAs was subsequently coated with hyaluronic acid (HA), a naturally occurring polymer, to mask the surface charge of PEI (HP/Si-D). In this procedure, DOX was first loaded into a scrambled siRNA and then condensed by PEI along with an antiautophagy siRNA. Due to its ability to preferentially connect with the cluster of differentiation 44 (CD44) receptor, which is overexpressed on a number of cancer cells,^250^ HA provided active targeting of the delivery system to tumor cells. By transfecting the cells with this system, the target cells’ autophagy level was downregulated. This resulted in a further reduction in ATP supply, which improved drug retention and cell cycle arrest. The findings, in particular, showed that autophagy and DOX combined effects caused synergistically stronger anticancer outcomes in vitro and in vivo than each treatment alone.^251^

Chitosan is a mucopolysaccharide that has a main amine in the glucosamine residue’s C-2 position, making it simple to be functionalized.^252^ Chitosan NPs are readily prepared, stable, biocompatible, and capable of controlling the release of active ingredients.^253^ In one study, gefitinib, a medication used to treat certain types of cancer, and short hairpin ATG-5 (shAtg-5) were delivered via a polymer-based delivery system that utilized chitosan nanoparticles. The findings demonstrated that the codelivery of gefitinib and shAtg-5 boosted cytotoxicity, significantly increased apoptosis by suppressing autophagy, and considerably reduced tumor growth.^254^

A plain sheet of carbon atoms forms the hexagonally, two-dimensional (2D) crystal structure known as graphene. The most significant graphene derivative is GO, which is the oxidized version of the material and has outstanding water processability, amphiphilicity, and surface functionalization capability.^255^ Stathmin1 gene, a key regulator of autophagy that has been discovered to be up-regulated in various malignancies,^256−258^ could also be repressed by miR-101.^259^ Based on that, a recent study based on the use of cationized graphene oxide (GO) as a delivery system to provide improved delivery of miR-101and enhanced photothermal therapy has been conducted. In this study GO surface has been functionalized with amine polyethylene glycol (PEG) and Poly-l-arginine (P-l-Arg) to improve particle stability and biocompatibility, as well as to facilitate the contact with target cells. The obtained results indicated that GO-PEG-(P-l-Arg) would be a promising targeted delivery strategy for miR-101 transfection that could suppress autophagy and convey thermal stress to trigger apoptotic cascades when combined with photothermal therapy.^260^ Another study demonstrated the use of GO functionalized with polyethylenimine, PEI, (PEI-GO) to codeliver Bcl-2 siRNA and DOX to cancer cells. The combination of GO and PEI was predicted to aid in the electrostatic adsorption of siRNA and the stacking of aromatic anticancer medications onto GO sheets. The findings highlighted that the codelivery of siRNA and DOX enhanced the efficiency of the cytotoxic drug by synergistically promoting apoptosis.^261^

Micelles are a family of amphiphilic copolymers and surfactants that function as core–shell nanocarriers.^262^ They help encapsulate hydrophobic chemotherapeutic medicines and hydrophilic gene drugs. Notably, micelles have great solubility, stability, and biodistribution, and because of their increased penetration and retention (EPR) effect, they can passively accumulate in tumor tissues.^263^ In a recent study, a peptide micelle system (Co-PMs) was developed using arginine and histidine copolymers to codeliver Beclin-1 siRNA and 6-maleimidocaproylhydrazone DOX derivative, DOX-EMCH. Histidine was shown to assist nanomicelles in escaping from endosomes through the proton sponge effect, whereas arginine was employed to compress RNA through electrostatic interactions. In accordance with in vitro findings, Co-PMs were successful in achieving siRNA endosomal escape, demonstrated greater cytotoxicity in PC3 cells than DOX alone, and consequently, increased the susceptibility of the cells to DOX. Furthermore, the Co-PMs demonstrated a 3 times stronger tumor inhibitory capacity in vivo compared to DOX or Beclin-1 siRNA therapy alone, indicating a considerable antitumor activity.^264^

In a recent study, a polymer-based micelle delivery system was developed to codeliver docetaxel (DTX) with ATG-7 siRNA, which inhibits autophagy, simultaneously in vitro and in vivo. Where it has been demonstrated that DTX induces autophagy, which is accountable for drug resistance. The hydrophobic drug, DTX, and the ATG-7 siRNA were enclosed in the synthesized PP6iRGD micelles. In order to create PP6iRGD, PEI was first reacted with the triblock copolymer Pluronic P123. The resultant compound, PP6, was then further coupled with the iRGD (CRGDK/RGPD/EC) peptide. The vehicle was given effective cationic characteristics for binding the gene due to the presence of PEI. While iRGD successfully targeted tumor by binding to integrins that are overexpressed on the endothelium of tumor arteries. Results revealed that ATG-7 knockdown reduced autophagy, which improved the effects of DTX treatment as seen by a considerable rise in cellular apoptosis.^265^ In a related study, a unique approach for treating breast cancer was demonstrated using codelivery of DTX and ATG-7 siRNA in a cross-linked, reducible, polypeptide micellar system. The core components of the system were lipoic acid (LA) and cytosol localization and internalization peptide 6 (CL), which may self-assemble into micelles and then be cross-linked to form cross-linked micelles. With exceptional biocompatibility and serum stability, CL is a novel cell-penetrating peptide (CPP) that may readily penetrate cellular membranes and transfer payload to the cytosol through using nonendosomal pathways. While LA has antioxidant properties since the human body naturally produces it. The findings demonstrated that ATG-7 siRNA might inhibit cells’ capacity to activate autophagy by silencing ATG-7 gene, hence reducing the survival of human MCF-7 cancer cells after chemotherapy. That was clear from the fact that the combined effects of DTX and siATG7 on cancer cells were 2.5 and 1.7 times greater than those of DTX alone in terms of cytotoxicity and apoptosis, respectively.^266^

Nonetheless, a peptide-based micellar delivery system has been developed to accomplish a codelivery of adenosine monophosphate-activated protein kinase (AMPK) activator narciclasine (Narc) with unc-51-like kinase 1 (ULK1) siRNA into HCC cells. As it has been established that activating AMPK regulates mTOR-dependent signaling pathways to inhibit HCC.^267^ While ULK1 knockdown inhibits autophagy and, as a result, increases apoptosis. The utilization of lipid-modified cell-penetrating peptides served as the foundation for the development of the self-assembling, biocompatible, pH-sensitive micellar system. A markedly disturbed autophagy was seen in tumor cells when the micelles were transfected into HCC cells in vitro. Furthermore, when the micelles were administered to mice with HCC xenografts, apoptosis was induced, tumor growth was decreased, and autophagy was prevented. These results demonstrate that the synergistic administration of Narc and siULK1 in biocompatible micelles can safely reduce tumor growth and autophagy.^268^

Liposomes are phospholipid vesicles that include aqueous gaps inside one or more concentric lipid bilayers. They are biocompatible and biodegradable, and they can recognize and target cancers.^269^ They can readily be coated with hydrophilic polymers, like PEG, to lengthen the in vivo circulation period.^270^ For instance, one study used PEGylated liposomes to deliver glyceraldehyde-3-phosphate dehydrogenase (GAPDH) siRNA and PTX simultaneously. As it has been demonstrated that blocking GAPDH will result in lower ATP levels, autophagy, and multidrug resistance (MDR) in hypoxic cancerous cells. The liposome delivery system included a cationic inner monolayer lipid vesicle in which GAPDH siRNA was inserted, a PEG-coated outer layer to shield the nanocarrier, and PTX sandwiched in between the two layers. Results obtained in vitro demonstrated that the designed approach had great specificity in GAPDH suppression and synergistically increased the cytotoxicity of PTX in tumor cells (HeLa and MCF-7) in a hypoxic environment. Additionally, in vivo studies indicated that the liposomes improved the PTX chemotherapeutic activity while also gradually increasing drug concentrations in tumors. When GAPDH siRNA was codelivered with PTX utilizing liposomes, tumor cells became more susceptible to PTX and thus treatment outcomes were improved.^271^

Nevertheless, other nanocarriers have been also utilized in delivering autophagy-related siRNAs and miRNAs into cancer cells. For example, cisplatin and Beclin-1 siRNA were simultaneously delivered to lung cancer cells using a peptide-based nanoprodrug delivery system. Three primary elements made up the delivery system. The prodrug complex (Pt(IV)-peptide-bis(pyrene)) was first created by conjugating tetravalent cisplatin (Pt(IV)) to a cationic peptide and then functionalizing it onto the hydrophobic polymer backbone. This cisplatin functionalization ensured great drug loading efficiency (up to 95%) and aided in the delivery of Beclin-1 siRNA into the cytosol by providing cationic charge for the complexation. To further strengthen and stabilize the system’s biocompatibility, the DSPE-PEG molecule was introduced. Finally, to provide targeted administration, cRGD was fixed onto the DSPE-PEG molecule. The system caused the Beclin-1 gene to be suppressed, hence blocking autophagy. As a result, the medicine’s effectiveness was improved, drug resistance was overcome, and tumor growth in a xenograft mice model was inhibited.^272^ Another investigation used lipid-coated calcium carbonate nanoparticles to deliver sorafenib and miR-375 in combination for the treatment of HCC. The lipid had a positive charge that was used for electrostatically complexing with the miRNA. With the help of this approach, autophagy has been successfully inhibited. According to in vitro data, the nanoparticles displayed high cytotoxicity and pH-dependent drug release. Whereas in vivo results indicated that the chemotherapeutic drug’s efficiency was greatly increased as demonstrated by a reduction in tumor sizes.^273^ In our current research group work, a “smart” nanocarrier has been developed as the carrier of LC3 siRNA to deliver combination of DOX and LC3 siRNA for cancer therapy in TNBC. The “smart” nanoparticle system was based on the use of FDA -approved β-cyclodextrin (β-CD) that consists of two sides: a primary side and a secondary side. Half of the primary side were functionalized with three different moieties: 2-(dimethylamino) ethyl methacrylate (DMAEMA) monomers, which are pH sensitive, hydrophobic hexyl methacrylate (HMA) and cationic trimethylaminoethyl methacrylate iodide (TMAEMA) monomers, while the other half was PEG bound. Results showed that this polymer was able to detect changes in the environmental pH, disrupt the endosomal membrane, and achieve electrostatic complexation between its cationic amine groups and the anionic phosphate groups of LC3 siRNA. We demonstrated that utilizing this delivery approach could efficiently suppress the autophagy-related gene LC3, inhibit cellular autophagy and exhibit improved anticancer effects. Furthermore, in the TNBC cell line, MDA-MB-231, coadministration of siLC3 and DOX was more effective than either agent alone in treating breast cancer. The inhibition of cell growth, colony formation, and migration in the cells demonstrated this. Furthermore, our combination caused an increase in the apoptotic population, as shown by an increase in the sub-G1 population, as well as induction in G2/M cell cycle arrest.

#### 3.2.2 Nanoparticles as Autophagy Inhibitors

Several NPs have the ability to modulate autophagy on their own, either by enhancing or suppressing the autophagic flux. Lysosomal dysfunction is the predominant way through which autophagy got manipulated since lysosomes are involved in the process’s final stages. Autophagosome accumulation is the main effect of this lysosome dysfunction. It has been shown that a range of NPs, including metal oxides, carbon nanomaterials, gold NPs, quantum dots, iron oxide NPs, graphene NPs, silver NPs (AgNPs), nanosized fullerene, and the nanosized fullerene derivative can cause autophagosome accumulation and evoke autophagy.^274−285^ Unfortunately, some of the studies mentioned above did not take into account the relationship between the number of autophagosomes and the rates at which they are produced and degraded at any given time. Since accumulation of autophagosomes may result from either autophagy blockage or autophagy stimulation. In this part, we highlighted a number of studies where cells exposed to naked NPs resulted in autophagy dysfunction.

Carbon nanotubes (CNTs) have several distinctive characteristics that allow them to appeal for usage in a variety of nanomedicine applications, including intravascular use. One study examined the impact of two systems of carbon nanomaterials on autophagy, particularly in cultured murine peritoneal macrophages. These systems were acid-functionalized single-walled carbon nanotubes (AF-SWCNTs) and graphene oxides (GOs). By examining autophagosomes, lysosomes, and the amount of p62, it has been demonstrated that both systems were capable of causing an autophagy blockage. p62 was dose-dependently accumulated by AF-SWCNTs and GOs, which indicates that autophagy is being inhibited. Additionally, AF-SWCNTs and GOs accumulated in macrophage lysosomes and caused lysosome membrane instability, which in turn resulted in an increase of autophagosomes, suggesting impaired autophagic breakdown.^286^ Another study examined the impact of exposure to carboxylated multiwalled carbon nanotubes (MWCNTs) on autophagy in human umbilical vein endothelial cells (HUVECs). It was shown that using MWCNTs led to an accumulation of autophagosomes that was brought on by the blocking of the autophagic flux instead of through the activation of autophagy. Measuring the expression of p62, when no decline in its level was seen, demonstrated this.^287^ In a different investigation, pristine multiwall carbon nanotubes (PMWCNT) demonstrated an inhibition in autophagic flux demonstrated by an induction in pulmonary autophagy accumulation in normal mouse lung as well as an elevation in the level of p62 expression.^288^ One study used graphite carbon nanofibers (GCNFs), a possible substitute for carbon nanotubes (CNT), to block autophagy, which caused the activation of apoptosis in human lung cancer cells. The capacity of GCNFs to destabilize lysosomes and produce ROS resulted in an accumulation of autophagosomes that blocked the autophagic flux.^289^

One study found that AgNPs might cause lysosomal degradation and autophagy disruption in HepG2 cells, which led to the induction of apoptosis. It has been demonstrated that AgNPs could disrupt the autophagy–lysosomal pathway, hence activating pro-inflammatory caspase-1-dependent signaling.^290^ In another study, utilizing AgNPs resulted in dysfunctional autophagy by impairing autophagosome-lysosome fusion. By evaluating LC3 conversion and the level of p62 expression, the suppression of autophagy was demonstrated. In which a rise in LC3-I to LC3-II conversion as well as a dose-dependent buildup of p62 expression have been observed.^291^

In another study, silica nanoparticles (SiNPs) were exposed to the HepG2 hepatocellular carcinoma cell line, and the influence on autophagy was examined. SiNPs have been found to have the potential to impede autophagic flux by affecting lysosome function. The findings demonstrated that SiNPs are being taken up by endocytosis and building up in lysosomes, resulting in an overload on lysosomes that causes their edema and malfunction. In turn, autophagosomes and lysosomes were blocked from fusing, which prevented the breakdown of autophagic cargo. This was emphasized by AO staining, which showed increased lysosomal membrane permeability and lysosome breakdown. Yet, the level of p62 expression has also been studied, and its time-dependent increment highlighted the disruption of autophagic flux.^232^

It is worth noting that the NPs’ shape influences how they affect the autophagy pathway, lysosome activity, and toxicity profiles of the nanoparticles. According to one study, tubular carbon nanoparticles (CNT) restricted the autophagy flow in RAW 264.7 murine macrophages as contrast to spherical carbon nanoparticles, whose use had no effect on autophagy. It has been demonstrated that CNT prevented the fusion of the autophagosome with the lysosome and imposed autophagy suppression as a result of lysosome dysfunction and subsequent downregulation of the expression of the SNAP-associated protein (SNAPIN), a crucial coordinator of late endocytic transport and lysosomal maturation.^292^ In a different study, the impact of the two types of gold nanoparticles most frequently utilized in biomedical fields—gold nanospheres and nanorods—on autophagy was examined. In contrast to what was seen when Au nanorods were used, it has been found that Au nanospheres impede the lysosomal function by accumulating in autolysosomes. Lysosome dysfunction caused autophagic flow to be inhibited, which was suggested by the accumulation of autophagosomes, as demonstrated by an increase in LC3-II and an increase in p62.^293^

It is also interesting to note that altering the lipid content of the autophagosomal or lysosomal membranes hinders autophagosome-lysosome fusion, which lowers autophagic activity. In one in vitro study, it was discovered that exposing autophagosomes and lysosomes to the potent β-CD derivative, methyl-β-cyclodextrin (MBCD), decreased the cholesterol levels in the membranes by 25 and 70%, respectively. This change in the content of the membranes led to a 40–50% decrease in their fusion efficiency, which had an impact on the overall activity of autophagy.^294^ In a different investigation, in vivo effects of another β-CD derivative 2-hydroxypropyl-β-cyclodextrin (CYCLO) were examined. It has been demonstrated that CYCLO delayed autophagosome maturation and impeded autophagosome-lysosome fusion by increasing the accumulation of LC3-II and p62, as well as a buildup of autophagosomes.^295^

Additionally, a “smart” nanoparticle based on the utilization of β-CD has been created, and its endosomal escape mechanism is accomplished by both DMAEMA, which expresses proton-sponge influence, and the hydrophobic moiety HMA, which tears up the endosome membranes.^296,297^ It is thought that as the “smart” nanoparticle enters the lysosomes and autophagosomes as well, it bursts their membranes via the same mechanism, interrupting the autophagy pathway.

## 4 Challenges and Future Perspectives

In our comprehensive review of autophagy modulation in cancer therapy, we aim to elucidate the benefits and drawbacks of various therapeutic strategies while exploring the potential of nanoparticles in regulating autophagy. Traditional drug agents targeting autophagy pathways, such as chloroquine and hydroxychloroquine, offer the advantage of established clinical use. However, their efficacy may be limited by off-target effects and dose-dependent toxicity.^298,299^ Gene silencing therapy, utilizing RNA interference techniques, presents a promising avenue for precise modulation of autophagy-related genes. Yet, challenges persist in achieving efficient delivery and avoiding immune responses. Nanoparticles, with their unique properties and tunable characteristics, offer a promising platform for targeted delivery of therapeutic agents, including RNA-based therapies, to cancer cells. By encapsulating drugs or genetic material, nanoparticles can enhance drug stability, prolong circulation time, and facilitate cellular uptake, potentially overcoming the limitations of conventional drug delivery methods. Moreover, nanoparticles can be engineered to modulate autophagy directly through various mechanisms, such as targeting autophagic machinery or signaling pathways. However, issues such as biocompatibility, biodistribution, and scalability remain to be addressed for widespread clinical translation.^300^ Despite these challenges, the versatility of nanoparticles holds great promise for advancing precision cancer therapies by enabling the timely and accurate regulation of autophagy in tumor cells. As research in this field progresses, continued efforts to optimize nanoparticle design and delivery strategies will be essential for realizing the full therapeutic potential of autophagy modulation in cancer treatment.

In conclusion, the intricate relationship between autophagy and cancer presents a dual role for this cellular process in tumorigenesis and treatment response. While autophagy serves as a crucial mechanism for maintaining cellular homeostasis and adapting to metabolic stress, its dysregulation can contribute to tumor progression and resistance to therapy. Our review has highlighted the therapeutic potential of autophagy inhibition in improving cancer treatment outcomes, emphasizing various strategies such as drug agents, gene silencing therapy, and nanoparticle-based approaches. Through targeting key autophagy-related genes and modulating the autophagic flux, these interventions hold promise for enhancing the efficacy of traditional cancer therapies and overcoming drug resistance mechanisms. However, challenges remain in understanding the precise mechanisms underlying the dual role of autophagy in cancer and optimizing the delivery and efficacy of autophagy-targeting agents.

Furthermore, we have provided a comprehensive overview of key points, mechanisms, and challenges in autophagy and cancer therapy in Table 2, which summarizes the current understanding and ongoing efforts in this field. This table serves as a valuable resource for researchers and clinicians seeking to elucidate the complex interplay between autophagy and cancer and develop novel therapeutic strategies to combat this disease effectively.

**Table 2:** Key Points, Mechanisms, and Challenges in Autophagy and Cancer Therapy

Key Points	Details
Challenges and Future Directions	**• Drug resistance**: Autophagy inhibition may lead to the development of resistance mechanisms in cancer cells.
Autophagy Mechanisms	**• Macroautophagy**: The primary pathway involved in autophagy, characterized by the formation of autophagosomes that engulf cellular components for degradation.
	**• Microautophagy**: Direct engulfment of cytoplasmic material by lysosomes.
	**• Chaperone-mediated autophagy**: Selective degradation of proteins recognized by chaperone proteins.

Role of Autophagy in Cancer	**• Dual nature**: Autophagy can both suppress and promote cancer, depending on tumor stage and context.
	**• Tumor suppression**: Delays tumor onset and slows tumor dissemination in early stages.
	**• Tumor promotion**: Promotes tumor development, spread, and resistance to anticancer drugs in advanced stages.

Specific Autophagy Inhibition Strategies	**• Autophagy inhibition**: Utilizing drugs like chloroquine (CQ) and hydroxychloroquine (HCQ) to block autophagy at various stages.
	**• Gene silencing therapy**: Employing RNA interference (RNAi) techniques targeting critical autophagy-related genes (ATGs).
	**• Nanoparticles**: Serving as delivery vehicles for genetic material (siRNA, miRNA) to inhibit autophagy or modulate autophagic flow.

Delivery Matters	**• Delivery challenges**: Overcoming hurdles in delivering RNA-based therapies due to degradation and cell membrane penetration issues.
	**• Optimizing nanoparticle design**: Engineering nanoparticles with optimal properties for efficient delivery and autophagy modulation.

## 5 Conclusion

A substantial body of current research indicates that autophagy inhibition has been reported to cause tumor cell death, generates inflammation in the tumor microenvironment and cancer-adjacent cells, which promote carcinogenesis. Autophagy, however, has also been linked to tumor spreading as it increases the aggressiveness of malignancy. Recently, autophagy has also been highlighted as a potential factor in the emergence and development of anticancer drug resistance. Autophagy is a crucial factor in cell death decisions. The ability of autophagy to trigger mitophagy and decrease the concentration of pro-apoptotic proteins in the cytosol are the most plausible explanations. CQ, HCQ, 3-MA, and BafA1 are among the drug agents that have been designed to prevent autophagy at its various phases. Far from autophagic medicines, other methods for blocking autophagy have also been explored. Gene silencing tools, specifically siRNA and miRNA, are being used to suppress autophagy by focusing on critical ATG genes implicated in the process. However, because siRNA and miRNA quickly degrade once they reach the bloodstream and because of their negative charge, which prevents them from entering cell membranes, various nanoparticles have been used to successfully deliver them into tumor cells. In addition to their function as carriers of genetic material, nanoparticles have also been designed to modulate autophagy on their own by inhibiting the autophagic flow through accumulating autophagosomes. Accordingly, as one recent review highlighted, numerous efforts and studies are currently being made in an effort to improve the effectiveness of cancer treatment by utilizing various strategies to block autophagy.

## References

[ref1] Amaravadi R. K.; Kimmelman A. C.; Debnath J. Targeting autophagy in cancer: Recent advances and future directions. Cancer Discovery 2019, 9, 1167–81. 10.1158/2159-8290.CD-19-0292. 31434711 PMC7306856

[ref2] Singh A.; Handa M.; Ruwali M.; Flora S. J. S.; Shukla R.; Kesharwani P. Nanocarrier mediated autophagy: An emerging trend for cancer therapy. Process Biochem. 2021, 109, 198–206. 10.1016/j.procbio.2021.07.011.

[ref3] Scrivo A.; Bourdenx M.; Pampliega O.; Cuervo A. M. Selective autophagy as a potential therapeutic target for neurodegenerative disorders. Lancet Neurol. 2018, 17, 802–15. 10.1016/S1474-4422(18)30238-2. 30129476 PMC6359907

[ref4] Shimizu S. Biological roles of alternative autophagy. Mol. Cells 2018, 41, 50–54. 10.14348/molcells.2018.2215. 29370693 PMC5792713

[ref5] Kaushik S.; Cuervo A. M. The coming of age of chaperone-mediated autophagy. Nat. Rev. Mol. Cell Biol. 2018, 19, 365–81. 10.1038/s41580-018-0001-6. 29626215 PMC6399518

[ref6] Menzies F. M.; Fleming A.; Caricasole A.; Bento C. F.; Andrews S. P.; Ashkenazi A.; Füllgrabe J.; Jackson A.; Jimenez Sanchez M.; Karabiyik C.; Licitra F.; Lopez Ramirez A.; Pavel M.; Puri C.; Renna M.; Ricketts T.; Schlotawa L.; Vicinanza M.; Won H.; Zhu Y.; Skidmore J.; Rubinsztein D. C. Autophagy and Neurodegeneration: Pathogenic Mechanisms and Therapeutic Opportunities. Neuron 2017, 93, 1015–34. 10.1016/j.neuron.2017.01.022. 28279350

[ref7] Chang S. J.; Ou-Yang F.; Tu H. P.; Lin C. H.; Huang S. H.; Kostoro J.; Hou M. F.; Chai C. Y.; Kwan A. L. Decreased expression of autophagy protein LC3 and stemness (CD44+/CD24-/low) indicate poor prognosis in triple-negative breast cancer. Hum. Pathol. 2016, 48, 48–55. 10.1016/j.humpath.2015.09.034. 26772398

[ref8] Panzarini E.; Dini L. Nanomaterial-induced autophagy: A new reversal MDR tool in cancer therapy?. Mol. Pharmaceutics 2014, 11, 2527–38. 10.1021/mp500066v. 24921216

[ref9] Harding T. M.; Morano K. A.; Scott S. V.; Klionsky D. J. Isolation and characterization of yeast mutants in the cytoplasm to vacuole protein targeting pathway. J. Cell Biol. 1995, 131, 591–602. 10.1083/jcb.131.3.591. 7593182 PMC2120622

[ref10] Deretic V.; Saitoh T.; Akira S. Autophagy in infection, inflammation and immunity. Nat. Rev. Immunol. 2013, 13, 722–37. 10.1038/nri3532. 24064518 PMC5340150

[ref11] Levine B.; Mizushima N.; Virgin H. W. Autophagy in immunity and inflammation. Nature 2011, 469, 323–35. 10.1038/nature09782. 21248839 PMC3131688

[ref12] Xu Y.; Yu H.; Qin H.; Kang J. S.; Yu C.; Zhong J.; Su J.; Li H. Y.; Sun L. K. Inhibition of autophagy enhances cisplatin cytotoxicity through endoplasmic reticulum stress in human cervical cancer cells. Cancer Lett. 2012, 314, 232–43. 10.1016/j.canlet.2011.09.034. 22019047

[ref13] Wang P.; Zhang L.; Chen Z.; Meng Z. MicroRNA targets autophagy in pancreatic cancer cells during cancer therapy. Autophagy 2013, 9, 2171–2. 10.4161/auto.26463. 24145177

[ref14] Kim J. S.; Bae G. E.; Kim K. H.; Lee S. Il; Chung C.; Lee D.; Lee T. H.; Kwon I. S.; Yeo M. K. Prognostic significance of LC3B and p62/SQSTM1 expression in gastric adenocarcinoma. Anticancer Res. 2019, 39, 6711–22. 10.21873/anticanres.13886. 31810936

[ref15] Mizushima N.; Yoshimori T.; Levine B. Methods in Mammalian Autophagy Research. Cell 2010, 140, 313–26. 10.1016/j.cell.2010.01.028. 20144757 PMC2852113

[ref16] Tanida I.; Ueno T.; Kominami E. LC3 conjugation system in mammalian autophagy. Int. J. Biochem. Cell Biol. 2004, 36, 2503–18. 10.1016/j.biocel.2004.05.009. 15325588 PMC7129593

[ref17] Mitroi D. N.; Karunakaran I.; Gräler M.; Saba J. D.; Ehninger D.; Ledesma M. D.; van Echten-Deckert G. SGPL1 (sphingosine phosphate lyase 1) modulates neuronal autophagy via phosphatidylethanolamine production. Autophagy 2017, 13, 885–99. 10.1080/15548627.2017.1291471. 28521611 PMC5446076

[ref18] Fogel A. I.; Dlouhy B. J.; Wang C.; Ryu S.-W.; Neutzner A.; Hasson S. A.; Sideris D. P.; Abeliovich H.; Youle R. J. Role of Membrane Association and Atg14-Dependent Phosphorylation in Beclin-1-Mediated Autophagy. Mol. Cell. Biol. 2013, 33, 3675–88. 10.1128/MCB.00079-13. 23878393 PMC3753860

[ref19] Zalckvar E.; Berissi H.; Mizrachy L.; Idelchuk Y.; Koren I.; Eisenstein M.; Sabanay H.; Pinkas-Kramarski R.; Kimchi A. DAP-kinase-mediated phosphorylation on the BH3 domain of beclin 1 promotes dissociation of beclin 1 from Bcl-XL and induction of autophagy. EMBO Rep. 2009, 10, 285–92. 10.1038/embor.2008.246. 19180116 PMC2658558

[ref20] Aparicio R.; Rana A.; Walker D. W. Upregulation of the Autophagy Adaptor p62/SQSTM1 Prolongs Health and Lifespan in Middle-Aged Drosophila. Cell Rep. 2019, 28, 1029–1040. 10.1016/j.celrep.2019.06.070. 31340141 PMC6688777

[ref21] Guo J. Y.; Teng X.; Laddha S. V.; Ma S.; Van Nostrand S. C.; Yang Y.; Khor S.; Chan C. S.; Rabinowitz J. D.; White E. Autophagy provides metabolic substrates to maintain energy charge and nucleotide pools in Ras-driven lung cancer cells. Genes Dev. 2016, 30, 1704–17. 10.1101/gad.283416.116. 27516533 PMC5002976

[ref22] Kuma A.; Mizushima N. Physiological role of autophagy as an intracellular recycling system: With an emphasis on nutrient metabolism. Semin. Cell Dev. Biol. 2010, 21, 683–90. 10.1016/j.semcdb.2010.03.002. 20223289

[ref23] Nah J.; Yuan J.; Jung Y. K. Autophagy in neurodegenerative diseases: From mechanism to therapeutic approach. Mol. Cells 2015, 38, 381–9. 10.14348/molcells.2015.0034. 25896254 PMC4443278

[ref24] Dikic I.; Elazar Z. Mechanism and medical implications of mammalian autophagy. Nat. Rev. Mol. Cell Biol. 2018, 19, 349–64. 10.1038/s41580-018-0003-4. 29618831

[ref25] Mowers E. E.; Sharifi M. N.; Macleod K. F. Autophagy in cancer metastasis. Oncogene 2017, 36, 1619–30. 10.1038/onc.2016.333. 27593926 PMC5337449

[ref26] Avivar-Valderas A.; Bobrovnikova-Marjon E.; Alan Diehl J.; Bardeesy N.; Debnath J.; Aguirre-Ghiso J. A. Regulation of autophagy during ECM detachment is linked to a selective inhibition of mTORC1 by PERK. Oncogene 2013, 32, 4932–40. 10.1038/onc.2012.512. 23160380 PMC3600386

[ref27] Li X.; He S.; Ma B. Autophagy and autophagy-related proteins in cancer. Mol. Cancer 2020, 19, 12 10.1186/s12943-020-1138-4. 31969156 PMC6975070

[ref28] Nurdinov N. LC3 and Beclin-1 as Markers of Autophagic Activity in Breast Cancer. Erciyes Med. J. 2020, 43, 333–336. 10.14744/etd.2020.99997.

[ref29] White E. The role for autophagy in cancer. J. Clin. Invest. 2015, 125, 42–6. 10.1172/JCI73941. 25654549 PMC4382247

[ref30] Long X.; Yan J.; Zhang Z.; Chang J.; He B.; Sun Y.; Liang Y. Autophagy-targeted nanoparticles for effective cancer treatment: advances and outlook. NPG Asia Mater. 2022, 14, 71 10.1038/s41427-022-00422-3.

[ref31] Galluzzi L.; Baehrecke E. H.; Ballabio A.; Boya P.; Bravo-San Pedro J. M.; Cecconi F.; Choi A. M.; Chu C. T.; Codogno P.; Colombo M. I.; Cuervo A. M.; Debnath J.; Deretic V.; Dikic I.; Eskelinen E.; Fimia G. M.; Fulda S.; Gewirtz D. A.; Green D. R.; Hansen M.; Harper J. W.; Jäättelä M.; Johansen T.; Juhasz G.; Kimmelman A. C.; Kraft C.; Ktistakis N. T.; Kumar S.; Levine B.; Lopez-Otin C.; Madeo F.; Martens S.; Martinez J.; Melendez A.; Mizushima N.; Münz C.; Murphy L. O.; Penninger J. M.; Piacentini M.; Reggiori F.; Rubinsztein D. C.; Ryan K. M.; Santambrogio L.; Scorrano L.; Simon A. K.; Simon H.; Simonsen A.; Tavernarakis N.; Tooze S. A.; Yoshimori T.; Yuan J.; Yue Z.; Zhong Q.; Kroemer G. Molecular definitions of autophagy and related processes. EMBO J. 2017, 36, 1811–36. 10.15252/embj.201796697. 28596378 PMC5494474

[ref32] Lin Y.-X.; Gao Y.-J.; Wang Y.; Qiao Z.-Y.; Fan G.; Qiao S.-L.; Zhang R.-X.; Wang L.; Wang H. pH-Sensitive Polymeric Nanoparticles with Gold(I) Compound Payloads Synergistically Induce Cancer Cell Death through Modulation of Autophagy. Mol. Pharmaceutics 2015, 12, 2869–78. 10.1021/acs.molpharmaceut.5b00060. 26101892

[ref33] Shakeel-u-Rehman; Rah B.; Lone S. H.; Rasool R. U.; Farooq S.; Nayak D.; Chikan N. A.; Chakraborty S.; Behl A.; Mondhe D. M.; Goswami A.; Bhat K. A. Design and Synthesis of Antitumor Heck-Coupled Sclareol Analogues: Modulation of BH3 Family Members by SS-12 in Autophagy and Apoptotic Cell Death. J. Med. Chem. 2015, 58, 3432–44. 10.1021/jm501942m. 25825934

[ref34] Salah F. S.; Ebbinghaus M.; Muley V. Y.; Zhou Z.; Al-Saadi K. R. D.; Pacyna-Gengelbach M.; O’sullivan G. A.; Betz H.; König R.; Wang Z. Q.; Bräuer R.; Petersen I. Tumor suppression in mice lacking GABARAP, an atg8/lc3 family member implicated in autophagy, is associated with alterations in cytokine secretion and cell death. Cell Death Dis. 2016, 7, e2205 10.1038/cddis.2016.93. 27124579 PMC4855672

[ref35] Qu X.; Yu J.; Bhagat G.; Furuya N.; Hibshoosh H.; Troxel A.; Rosen J.; Eskelinen E. L.; Mizushima N.; Ohsumi Y.; Cattoretti G.; Levine B. Promotion of tumorigenesis by heterozygous disruption of the beclin 1 autophagy gene. J. Clin. Invest. 2003, 112, 1809–20. 10.1172/JCI20039. 14638851 PMC297002

[ref36] Takahashi Y.; Coppola D.; Matsushita N.; Cualing H. D.; Sun M.; Sato Y.; Liang C.; Jung J. U.; Cheng J. Q.; Mulé J. J.; Pledger W. J.; Wang H. G. Bif-1 interacts with Beclin 1 through UVRAG and regulates autophagy and tumorigenesis. Nat. Cell Biol. 2007, 9, 1142–51. 10.1038/ncb1634. 17891140 PMC2254521

[ref37] Takamura A.; Komatsu M.; Hara T.; Sakamoto A.; Kishi C.; Waguri S.; Eishi Y.; Hino O.; Tanaka K.; Mizushima N. Autophagy-deficient mice develop multiple liver tumors. Genes Dev. 2011, 25, 795–800. 10.1101/gad.2016211. 21498569 PMC3078705

[ref38] An C. H.; Kim M. S.; Yoo N. J.; Park S. W.; Lee S. H. Mutational and expressional analyses of ATG5, an autophagy-related gene, in gastrointestinal cancers. Pathol. Res. Pract. 2011, 207, 433–7. 10.1016/j.prp.2011.05.002. 21664058

[ref39] Amaravadi R.; Kimmelman A. C.; White E. Recent insights into the function of autophagy in cancer. Genes Dev. 2016, 30, 1913–30. 10.1101/gad.287524.116. 27664235 PMC5066235

[ref40] Russell R. C.; Yuan H. X.; Guan K. L. Autophagy regulation by nutrient signaling. Cell Res. 2014, 24, 42–57. 10.1038/cr.2013.166. 24343578 PMC3879708

[ref41] He L.; Zhang J.; Zhao J.; Ma N.; Kim S. W.; Qiao S.; Ma X. Autophagy: The last defense against cellular nutritional stress. Adv. Nutr. 2018, 9, 493–504. 10.1093/advances/nmy011. 30032222 PMC6054220

[ref42] Barnard R. A.; Regan D. P.; Hansen R. J.; Maycotte P.; Thorburn A.; Gustafson D. L. Autophagy inhibition delays early but not late-stage metastatic disease. J. Pharmacol. Exp. Ther. 2016, 358, 282–93. 10.1124/jpet.116.233908. 27231155 PMC4959099

[ref43] Liu E. Y.; Ryan K. M. Autophagy and cancer - issues we need to digest. J. Cell Sci. 2012, 125, 2349–58. 10.1242/jcs.093708. 22641689

[ref44] Wolf J.; Dewi D. L.; Fredebohm J.; Müller-Decker K.; Flechtenmacher C.; Hoheisel J. D.; Boettcher M. A mammosphere formation RNAi screen reveals that ATG4A promotes a breast cancer stem-like phenotype. Breast Cancer Res. 2013, 15, R109 10.1186/bcr3576. 24229464 PMC3978845

[ref45] Li J.-M.; Yu H.; Deng K.; Wang Q.; Li K.-H.; Huang S.-W. Liensinine Inhibits Mitophagy to Potentiate Mitochondria-Targeting Artemisinin-Loaded Polymer Nanostructures for Breast Cancer Therapy. ACS Appl. Nano Mater. 2023, 6, 22209–21. 10.1021/acsanm.3c04441.

[ref46] Taylor M. A.; Das B. C.; Ray S. K. Targeting autophagy for combating chemoresistance and radioresistance in glioblastoma. Apoptosis 2018, 23, 563–75. 10.1007/s10495-018-1480-9. 30171377 PMC6193815

[ref47] Maiuri M. C.; Kroemer G. Therapeutic modulation of autophagy: which disease comes first?. Cell Death Differ. 2019, 26, 680–9. 10.1038/s41418-019-0290-0. 30728461 PMC6460393

[ref48] Levy J. M. M.; Towers C. G.; Thorburn A. Targeting autophagy in cancer. Nat. Rev. Cancer 2017, 17, 528–42. 10.1038/nrc.2017.53. 28751651 PMC5975367

[ref49] Sooro M. A.; Zhang N.; Zhang P. Targeting EGFR-mediated autophagy as a potential strategy for cancer therapy. Int. J. Cancer 2018, 143, 2116–25. 10.1002/ijc.31398. 29574749

[ref50] Denisenko T. V.; Pivnyuk A. D.; Zhivotovsky B. P53-autophagy-metastasis link. Cancers (Basel) 2018, 10, 148 10.3390/cancers10050148. 29783720 PMC5977121

[ref51] Liu F.; Lee J. Y.; Wei H.; Tanabe O.; Engel J. D.; Morrison S. J.; Guan J. L. FIP200 is required for the cell-autonomous maintenance of fetal hematopoietic stem cells. Blood 2010, 116, 4806–14. 10.1182/blood-2010-06-288589. 20716775 PMC3321744

[ref52] Wang X.; Wang P.; Zhu Y.; Li S. Correlation between autophagy related genes expression and clinical features in carcinogenesis of oral squamous cell carcinoma. Int. J. Clin. Exp. Pathol. 2016, 9, 6307–16.

[ref53] Wei H.; Wei S.; Gan B.; Peng X.; Zou W.; Guan J. L. Suppression of autophagy by FIP200 deletion inhibits mammary tumorigenesis. Genes Dev. 2011, 25, 1510–27. 10.1101/gad.2051011. 21764854 PMC3143941

[ref54] Yang S.; Wang X.; Contino G.; Liesa M.; Sahin E.; Ying H.; Bause A.; Li Y.; Stommel J. M.; Dell'Antonio G.; Mautner J.; Tonon G.; Haigis M.; Shirihai O. S.; Doglioni C.; Bardeesy N.; Kimmelman A. C. Pancreatic cancers require autophagy for tumor growth. Genes Dev. 2011, 25, 717–29. 10.1101/gad.2016111. 21406549 PMC3070934

[ref55] Guo J. Y.; Chen H. Y.; Mathew R.; Fan J.; Strohecker A. M.; Karsli-Uzunbas G.; Kamphorst J. J.; Chen G.; Lemons J. M. S.; Karantza V.; Coller H. A.; DiPaola R. S.; Gelinas C.; Rabinowitz J. D.; White E. Activated Ras requires autophagy to maintain oxidative metabolism and tumorigenesis. Genes Dev. 2011, 25, 460–70. 10.1101/gad.2016311. 21317241 PMC3049287

[ref56] Yang A.; Kimmelman A. C. Inhibition of autophagy attenuates pancreatic cancer growth independent of TP53/TRP53 status. Autophagy 2014, 10, 1683–4. 10.4161/auto.29961. 25046107 PMC4206544

[ref57] Choi J.; Jung W.; Koo J. S. Expression of autophagy-related markers beclin-1, light chain 3A, light chain 3B and p62 according to the molecular subtype of breast cancer. Histopathology 2013, 62, 275–86. 10.1111/his.12002. 23134379

[ref58] Hamurcu Z.; Delibaşı N.; Geçene S.; Şener E. F.; Dönmez-Altuntaş H.; Özkul Y.; Canatan H.; Ozpolat B. Targeting LC3 and Beclin-1 autophagy genes suppresses proliferation, survival, migration and invasion by inhibition of Cyclin-D1 and uPAR/Integrin β1/ Src signaling in triple negative breast cancer cells. J. Cancer Res. Clin. Oncol. 2018, 144, 415–30. 10.1007/s00432-017-2557-5. 29288363 PMC11813384

[ref59] Yang A.; Kimmelman A. C. Inhibition of autophagy attenuates pancreatic cancer growth independent of TP53/TRP53 status. Autophagy 2014, 10, 1683–4. 10.4161/auto.29961. 25046107 PMC4206544

[ref60] Kim M. J.; Min Y.; Im J. S.; Son J.; Lee J. S.; Lee K. Y. P62 is negatively implicated in the TRAF6-BECN1 signaling axis for autophagy activation and cancer progression by toll-like receptor 4 (TLR4). Cells 2020, 9, 1142 10.3390/cells9051142. 32384667 PMC7290749

[ref61] Chen M. Y.; Yadav V. K.; Chu Y. C.; Ong J. R.; Huang T. Y.; Lee K. F.; Lee K. H.; Yeh C. T.; Lee W. H. Hydroxychloroquine (Hcq) modulates autophagy and oxidative dna damage stress in hepatocellular carcinoma to overcome sorafenib resistance via tlr9/sod1/hsa-mir-30a5p/beclin-1 axis. Cancers (Basel) 2021, 13, 3227 10.3390/cancers13133227. 34203465 PMC8267639

[ref62] Kang R.; Tang D.; Livesey K. M.; Schapiro N. E.; Lotze M. T.; Zeh H. J. The Receptor for Advanced Glycation End-Products (RAGE) Protects Pancreatic Tumor Cells Against Oxidative Injury. Antioxid. Redox Signal. 2011, 15, 2175–84. 10.1089/ars.2010.3378. 21126167 PMC3166176

[ref63] Roedig H.; Damiescu R.; Zeng-Brouwers J.; Kutija I.; Trebicka J.; Wygrecka M.; Schaefer L. Danger matrix molecules orchestrate CD14/CD44 signaling in cancer development. Semin. Cancer Biol. 2020, 62, 31–47. 10.1016/j.semcancer.2019.07.026. 31412297

[ref64] Pelosse M.; Cottet-Rousselle C.; Bidan C. M.; Dupont A.; Gupta K.; Berger I.; Schlattner U. Synthetic energy sensor AMPfret deciphers adenylate-dependent AMPK activation mechanism. Nat. Commun. 2019, 10, 1038 10.1038/s41467-019-08938-z. 30833561 PMC6399333

[ref65] Wang C.; Wang H.; Zhang D.; Luo W.; Liu R.; Xu D.; Diao L.; Liao L.; Liu Z. Phosphorylation of ULK1 affects autophagosome fusion and links chaperone-mediated autophagy to macroautophagy. Nat. Commun. 2018, 9, 3492 10.1038/s41467-018-05449-1. 30154410 PMC6113293

[ref66] Mickymaray S.; Alfaiz F. A.; Paramasivam A.; Veeraraghavan V. P.; Periadurai N. D.; Surapaneni K. M.; Niu G. Rhaponticin suppresses osteosarcoma through the inhibition of PI3K-Akt-mTOR pathway. Saudi J. Biol. Sci. 2021, 28, 3641–9. 10.1016/j.sjbs.2021.05.006. 34220214 PMC8241634

[ref67] Abreu S.; Kriegenburg F.; Gómez-Sánchez R.; Mari M.; Sánchez-Wandelmer J.; Skytte Rasmussen M.; Soares Guimarães R.; Zens B.; Schuschnig M.; Hardenberg R.; Peter M.; Johansen T.; Kraft C.; Martens S.; Reggiori F. Conserved Atg8 recognition sites mediate Atg4 association with autophagosomal membranes and Atg8 deconjugation. EMBO Rep. 2017, 18, 765–80. 10.15252/embr.201643146. 28330855 PMC5412903

[ref68] Liu W.; Glunde K.; Bhujwalla Z. M.; Raman V.; Sharma A.; Phang J. M. Proline oxidase promotes tumor cell survival in hypoxic tumor microenvironments. Cancer Res. 2012, 72, 3677–86. 10.1158/0008-5472.CAN-12-0080. 22609800 PMC3399032

[ref69] Yang H. L.; Liu H. W.; Shrestha S.; Thiyagarajan V.; Huang H. C.; Hseu Y. C. Antrodia Salmonea Induces Apoptosis and Enhances Cytoprotective Autophagy in Colon Cancer Cells. Aging (Albany. NY). 2021, 13, 15964–89. 10.18632/aging.203019. 34031264 PMC8266357

[ref70] Choi H. D.; Kim K.-Y.; Park K. Il; Kim S.-H.; Park S.-G.; Yu S.-N.; Kim Y.-W.; Kim D. S.; Chung K. T.; Ahn S.-C. Dual role of reactive oxygen species in autophagy and apoptosis induced by compound PN in prostate cancer cells. Mol. Cell. Toxicol. 2021, 17, 41–50. 10.1007/s13273-020-00107-4.

[ref71] Sivaprasad U.; Basu A. Inhibition of ERK attenuates autophagy and potentiates tumour necrosis factor-α-induced cell death in MCF-7 cells. J. Cell. Mol. Med. 2008, 12, 1265–71. 10.1111/j.1582-4934.2008.00282.x. 18266953 PMC3865671

[ref72] Ahn J.-H.; Jegal H.; Choi M.-S.; Kim S.; Park S.-M.; Ahn J.; Han H.-Y.; Cho H.-S.; Yoon S.; Oh J.-H. TNFα enhances trovafloxacin-induced in vitro hepatotoxicity by inhibiting protective autophagy. Toxicol. Lett. 2021, 342, 73–84. 10.1016/j.toxlet.2021.02.009. 33609687

[ref73] New J.; Arnold L.; Ananth M.; Alvi S.; Thornton M.; Werner L.; Tawfik O.; Dai H.; Shnayder Y.; Kakarala K.; Tsue T. T.; Girod D. A.; Ding W. X.; Anant S.; Thomas S. M. Secretory autophagy in cancer-associated fibroblasts promotes head and neck cancer progression and offers a novel therapeutic target. Cancer Res. 2017, 77, 6679–91. 10.1158/0008-5472.CAN-17-1077. 28972076 PMC5712244

[ref74] Cotzomi-Ortega I.; Rosas-Cruz A.; Ramírez-Ramírez D.; Reyes-Leyva J.; Rodriguez-Sosa M.; Aguilar-Alonso P.; Maycotte P. Autophagy inhibition induces the secretion of macrophage migration inhibitory factor (MIF) with autocrine and paracrine effects on the promotion of malignancy in breast cancer. Biology (Basel) 2020, 9, 20 10.3390/biology9010020. 31963754 PMC7169388

[ref75] Macintosh R. L.; Timpson P.; Thorburn J.; Anderson K. I.; Thorburn A.; Ryan K. M. Inhibition of autophagy impairs tumor cell invasion in an organotypic model. Cell Cycle 2012, 11, 2022–9. 10.4161/cc.20424. 22580450 PMC3359125

[ref76] Peng Y.-F.; Shi Y.-H.; Ding Z.-B.; Ke A.-W.; Gu C.-Y.; Hui B.; Zhou J.; Qiu S.-J.; Dai Z.; Fan J. Autophagy inhibition suppresses pulmonary metastasis of HCC in mice via impairing anoikis resistance and colonization of HCC cells. Autophagy 2013, 9, 2056–68. 10.4161/auto.26398. 24157892

[ref77] Klemm F.; Joyce J. A. Microenvironmental regulation of therapeutic response in cancer. Trends Cell Biol. 2015, 25, 198–213. 10.1016/j.tcb.2014.11.006. 25540894 PMC5424264

[ref78] Kenific C. M.; Thorburn A.; Debnath J. Autophagy and metastasis: Another double-edged sword. Curr. Opin. Cell Biol. 2010, 22, 241–5. 10.1016/j.ceb.2009.10.008. 19945838 PMC2854304

[ref79] Mowers E. E.; Sharifi M. N.; Macleod K. F. Functions of autophagy in the tumor microenvironment and cancer metastasis. FEBS J. 2018, 285, 1751–66. 10.1111/febs.14388. 29356327 PMC5992019

[ref80] Galavotti S.; Bartesaghi S.; Faccenda D.; Shaked-Rabi M.; Sanzone S.; McEvoy A.; Dinsdale D.; Condorelli F.; Brandner S.; Campanella M.; Grose R.; Jones C.; Salomoni P. The autophagy-associated factors DRAM1 and p62 regulate cell migration and invasion in glioblastoma stem cells. Oncogene 2013, 32, 699–712. 10.1038/onc.2012.111. 22525272

[ref81] Han C.; Sun B.; Wang W.; Cai W.; Lou D.; Sun Y.; Zhao X. Overexpression of microtubule-associated protein-1 light chain 3 is associated with melanoma metastasis and vasculogenic mimicry. Tohoku J. Exp. Med. 2011, 223, 243–51. 10.1620/tjem.223.243. 21415575

[ref82] Zhao H.; Yang M.; Zhao J.; Wang J.; Zhang Y.; Zhang Q. High expression of LC3B is associated with progression and poor outcome in triple-negative breast cancer. Med. Oncol. 2013, 30, 475 10.1007/s12032-013-0475-1. 23371253

[ref83] Lazova R.; Camp R. L.; Klump V.; Siddiqui S. F.; Amaravadi R. K.; Pawelek J. M. Punctate LC3B expression is a common feature of solid tumors and associated with proliferation, metastasis, and poor outcome. Clin. Cancer Res. 2012, 18, 370–9. 10.1158/1078-0432.CCR-11-1282. 22080440 PMC4825867

[ref84] Hashemi-Sadraei N.; Müller-Greven G. M.; Abdul-Karim F. W.; Ulasov I.; Downs-Kelly E.; Burgett M. E.; Lauko A.; Qadan M. A.; Weil R. J.; Ahluwalia M. S.; Du L.; Prayson R. A.; Chao S. T.; Budd T. G.; Barnholtz-Sloan J.; Nowacki A. S.; Keri R. A.; Gladson C. L. Expression of LC3B and FIP200/Atg17 in brain metastases of breast cancer. J. Neurooncol. 2018, 140, 237–48. 10.1007/s11060-018-2959-5. 30094720

[ref85] Zhou J.; Hang D.; Jiang Y.; Chen J.; Han J.; Zhou W.; Jin G.; Ma H.; Dai J. Evaluation of genetic variants in autophagy pathway genes as prognostic biomarkers for breast cancer. Gene 2017, 627, 549–55. 10.1016/j.gene.2017.06.053. 28669927

[ref86] Yuzhalin A. E.; Yu D. Brain metastasis organotropism. Cold Spring Harb. Perspect. Med. 2020, 10, a037242 10.1101/cshperspect.a037242. 31548224 PMC7197417

[ref87] Peng Y.-F.; Shi Y.-H.; Shen Y.-H.; Ding Z.-B.; Ke A.-W.; Zhou J.; Qiu S.-J.; Fan J. Promoting Colonization in Metastatic HCC Cells by Modulation of Autophagy. PLoS One 2013, 8, e74407 10.1371/journal.pone.0074407. 24058558 PMC3772859

[ref88] Chittaranjan S.; Bortnik S.; Dragowska W. H.; Xu J.; Abeysundara N.; Leung A.; Go N. E.; DeVorkin L.; Weppler S. A.; Gelmon K.; Yapp D. T.; Bally M. B.; Gorski S. M. Autophagy inhibition augments the anticancer effects of epirubicin treatment in anthracycline-sensitive and -resistant triple-negative breast cancer. Clin. Cancer Res. 2014, 20, 3159–73. 10.1158/1078-0432.CCR-13-2060. 24721646

[ref89] Grasso S.; Pereira G. J. S.; Palmeira-dos-Santos C.; Calgarotto A. K.; Martínez-Lacaci I.; Ferragut J. A.; Smaili S. S.; Bincoletto C. Autophagy regulates Selumetinib (AZD6244) induced-apoptosis in colorectal cancer cells. Eur. J. Med. Chem. 2016, 122, 611–8. 10.1016/j.ejmech.2016.06.043. 27448918

[ref90] Palmeira-Dos-Santos C.; Pereira G. J. S.; Barbosa C. M. V.; Jurkiewicz A.; Smaili S. S.; Bincoletto C. Comparative study of autophagy inhibition by 3MA and CQ on Cytarabine-induced death of leukaemia cells. J. Cancer Res. Clin. Oncol. 2014, 140, 909–20. 10.1007/s00432-014-1640-4. 24659340 PMC11824056

[ref91] Xiao X.; Wang W.; Li Y.; Yang D.; Li X.; Shen C.; Liu Y.; Ke X.; Guo S.; Guo Z. HSP90AA1-mediated autophagy promotes drug resistance in osteosarcoma. J. Exp. Clin. Cancer Res. 2018, 37, 201 10.1186/s13046-018-0880-6. 30153855 PMC6114771

[ref92] Chen Z.; Jiang Q.; Zhu P.; Chen Y.; Xie X.; Du Z.; Jiang L.; Tang W. NPRL2 enhances autophagy and the resistance to Everolimus in castration-resistant prostate cancer. Prostate 2019, 79, 44–53. 10.1002/pros.23709. 30178500

[ref93] O’Reilly E. A.; Gubbins L.; Sharma S.; Tully R.; Guang M. H. Z.; Weiner-Gorzel K.; McCaffrey J.; Harrison M.; Furlong F.; Kell M.; McCann A. The fate of chemoresistance in triple negative breast cancer (TNBC). BBA Clin. 2015, 3, 257–75. 10.1016/j.bbacli.2015.03.003. 26676166 PMC4661576

[ref94] Aydinlik S.; Erkisa M.; Cevatemre B.; Sarimahmut M.; Dere E.; Ari F.; Ulukaya E. Enhanced cytotoxic activity of doxorubicin through the inhibition of autophagy in triple negative breast cancer cell line. Biochim. Biophys. Acta - Gen. Subj. 2017, 1861, 49–57. 10.1016/j.bbagen.2016.11.013. 27842219

[ref95] Saha T. LAMP2A overexpression in breast tumors promotes cancer cell survival via chaperone-mediated autophagy. Autophagy 2012, 8, 1643–56. 10.4161/auto.21654. 22874552 PMC3494593

[ref96] Guo B.; Tam A.; Santi S. A.; Parissenti A. M. Role of autophagy and lysosomal drug sequestration in acquired resistance to doxorubicin in MCF-7 cells. BMC Cancer 2016, 16, 762 10.1186/s12885-016-2790-3. 27687594 PMC5043608

[ref97] Kumar A.; Singh U. K.; Chaudhary A. Targeting autophagy to overcome drug resistance in cancer therapy. Future Med. Chem. 2015, 7, 1535–42. 10.4155/fmc.15.88. 26334206

[ref98] Aredia F.; Scovassi A. I. Manipulation of autophagy in cancer cells: an innovative strategy to fight drug resistance. Future Med. Chem. 2013, 5, 1009–21. 10.4155/fmc.13.85. 23734684

[ref99] Park J.-H.; Kim K. P.; Ko J.-J.; Park K.-S. PI3K/Akt/mTOR activation by suppression of ELK3 mediates chemosensitivity of MDA-MB-231 cells to doxorubicin by inhibiting autophagy. Biochem. Biophys. Res. Commun. 2016, 477, 277–82. 10.1016/j.bbrc.2016.06.057. 27301639

[ref100] Gao A. M.; Zhang X. Y.; Hu J. N.; Ke Z. P. Apigenin sensitizes hepatocellular carcinoma cells to doxorubic through regulating miR-520b/ATG7 axis. Chem. Biol. Interact. 2018, 280, 45–50. 10.1016/j.cbi.2017.11.020. 29191453

[ref101] Lu S.; Yao Y.; Xu G.; Zhou C.; Zhang Y.; Sun J.; Jiang R.; Shao Q.; Chen Y. CD24 regulates sorafenib resistance via activating autophagy in hepatocellular carcinoma. Cell Death Dis. 2018, 9, 646 10.1038/s41419-018-0681-z. 29844385 PMC5974417

[ref102] Jaidee R.; Kongpetch S.; Senggunprai L.; Prawan A.; Kukongviriyapan U.; Kukongviriyapan V. Phenformin inhibits proliferation, invasion, and angiogenesis of cholangiocarcinoma cells via AMPK-mTOR and HIF-1A pathways. Naunyn. Schmiedebergs. Arch. Pharmacol. 2020, 393, 1681–90. 10.1007/s00210-020-01885-3. 32383028

[ref103] Hou Y. J.; Dong L. W.; Tan Y. X.; Yang G. Z.; Pan Y. F.; Li Z.; Tang L.; Wang M.; Wang Q.; Wang H. Y. Inhibition of active autophagy induces apoptosis and increases chemosensitivity in cholangiocarcinoma. Lab. Investig. 2011, 91, 1146–57. 10.1038/labinvest.2011.97. 21647092

[ref104] Fitzwalter B. E.; Towers C. G.; Sullivan K. D.; Andrysik Z.; Hoh M.; Ludwig M.; O’Prey J.; Ryan K. M.; Espinosa J. M.; Morgan M. J.; Thorburn A. Autophagy Inhibition Mediates Apoptosis Sensitization in Cancer Therapy by Relieving FOXO3a Turnover. Dev. Cell 2018, 44, 555–565. 10.1016/j.devcel.2018.02.014. 29533771 PMC5866042

[ref105] Mariño G.; Niso-Santano M.; Baehrecke E. H.; Kroemer G. Self-consumption: The interplay of autophagy and apoptosis. Nat. Rev. Mol. Cell Biol. 2014, 15, 81–94. 10.1038/nrm3735. 24401948 PMC3970201

[ref106] Gump J. M.; Staskiewicz L.; Morgan M. J.; Bamberg A.; Riches D. W. H.; Thorburn A. Autophagy variation within a cell population determines cell fate through selective degradation of Fap-1. Nat. Cell Biol. 2014, 16, 47–54. 10.1038/ncb2886. 24316673 PMC3876036

[ref107] Guadamillas M. C.; Cerezo A.; del Pozo M. A. Overcoming anoikis - pathways to anchorageindependent growth in cancer. J. Cell Sci. 2011, 124, 3189–97. 10.1242/jcs.072165. 21940791

[ref108] Perera R. M.; Stoykova S.; Nicolay B. N.; Ross K. N.; Fitamant J.; Boukhali M.; Lengrand J.; Deshpande V.; Selig M. K.; Ferrone C. R.; Settleman J.; Stephanopoulos G.; Dyson N. J.; Zoncu R.; Ramaswamy S.; Haas W.; Bardeesy N. Transcriptional control of autophagy-lysosome function drives pancreatic cancer metabolism. Nature 2015, 524, 361–5. 10.1038/nature14587. 26168401 PMC5086585

[ref109] Guo J. Y.; White E. Autophagy is required for mitochondrial function, lipid metabolism, growth, and fate of KRASG12D-driven lung tumors. Autophagy 2013, 9, 1636–8. 10.4161/auto.26123. 23959381 PMC5424446

[ref110] Vera-Ramirez L.; Vodnala S. K.; Nini R.; Hunter; Green J. E. Autophagy promotes the survival of dormant breast cancer cells and metastatic tumour recurrence. Nat. Commun. 2018, 9, 1944 10.1038/s41467-018-04070-6. 29789598 PMC5964069

[ref111] Carew J. S.; Espitia C. M.; Esquivel J. A.; Mahalingam D.; Kelly K. R.; Reddy G.; Giles F. J.; Nawrocki S. T. Lucanthone is a novel inhibitor of autophagy that induces cathepsin D-mediated apoptosis. J. Biol. Chem. 2011, 286, 6602–13. 10.1074/jbc.M110.151324. 21148553 PMC3057822

[ref112] Xi G.; Hu X.; Wu B.; Jiang H.; Young C. Y. F.; Pang Y.; Yuan H. Autophagy inhibition promotes paclitaxel-induced apoptosis in cancer cells. Cancer Lett. 2011, 307, 141–8. 10.1016/j.canlet.2011.03.026. 21511395

[ref113] Frankel L. B.; Lund A. H. MicroRNA regulation of autophagy. Carcinogenesis 2012, 33, 2018–25. 10.1093/carcin/bgs266. 22902544

[ref114] Yang A.; Herter-Sprie G.; Zhang H.; Lin E. Y.; Biancur D.; Wang X.; Deng J.; Hai J.; Yang S.; Wong K. K.; Kimmelman A. C. Autophagy sustains pancreatic cancer growth through both cell-autonomous and nonautonomous mechanisms. Cancer Discovery 2018, 8, 276–87. 10.1158/2159-8290.CD-17-0952. 29317452 PMC5835190

[ref115] Yu Y.; Yang L.; Zhao M.; Zhu S.; Kang R.; Vernon P.; Tang D.; Cao L. Targeting microRNA-30a-mediated autophagy enhances imatinib activity against human chronic myeloid leukemia cells. Leukemia 2012, 26, 1752–60. 10.1038/leu.2012.65. 22395361

[ref116] Morel E.; Mehrpour M.; Botti J.; Dupont N.; Hamai A.; Nascimbeni A. C.; Codogno P. Autophagy: A druggable process. Annu. Rev. Pharmacol. Toxicol. 2017, 57, 375–98. 10.1146/annurev-pharmtox-010716-104936. 28061686

[ref117] Cook K. L.; Wärri A.; Soto-Pantoja D. R.; Clarke P. A. G.; Cruz M. I.; Zwart A.; Clarke R. Hydroxychloroquine inhibits autophagy to potentiate antiestrogen responsiveness in ER+ breast cancer. Clin. Cancer Res. 2014, 20, 3222–32. 10.1158/1078-0432.CCR-13-3227. 24928945 PMC4073207

[ref118] Lee S. W.; Kim H. K.; Lee N. H.; Yi H. Y.; Kim H. S.; Hong S. H.; Hong Y. K.; Joe Y. A. The synergistic effect of combination Temozolomide and chloroquine treatment is dependent on autophagy formation and p53 status in glioma cells. Cancer Lett. 2015, 360, 195–204. 10.1016/j.canlet.2015.02.012. 25681668

[ref119] Sasaki K.; Tsuno N. H.; Sunami E.; Tsurita G.; Kawai K.; Okaji Y.; Nishikawa T.; Shuno Y.; Hongo K.; Hiyoshi M.; Kaneko M.; Kitayama J.; Takahashi K.; Nagawa H. Chloroquine potentiates the anti-cancer effect of 5-fluorouracil on colon cancer cells. BMC Cancer 2010, 10, 370 10.1186/1471-2407-10-370. 20630104 PMC2914703

[ref120] Abdel-Aziz A. K.; Shouman S.; El-Demerdash E.; Elgendy M.; Abdel-Naim A. B. Chloroquine synergizes sunitinib cytotoxicity via modulating autophagic, apoptotic and angiogenic machineries. Chem. Biol. Interact. 2014, 217, 28–40. 10.1016/j.cbi.2014.04.007. 24751611

[ref121] Selvakumaran M.; Amaravadi R. K.; Vasilevskaya I. A.; O’Dwyer P. J. Autophagy inhibition sensitizes colon cancer cells to antiangiogenic and cytotoxic therapy. Clin. Cancer Res. 2013, 19, 2995–3007. 10.1158/1078-0432.CCR-12-1542. 23461901

[ref122] Schrezenmeier E.; Dörner T. Mechanisms of action of hydroxychloroquine and chloroquine: implications for rheumatology. Nat. Rev. Rheumatol. 2020, 16, 155–66. 10.1038/s41584-020-0372-x. 32034323

[ref123] Ben-Zvi I.; Kivity S.; Langevitz P.; Shoenfeld Y. Hydroxychloroquine: From malaria to autoimmunity. Clin. Rev. Allergy Immunol. 2012, 42, 145–53. 10.1007/s12016-010-8243-x. 21221847 PMC7091063

[ref124] D’alessandro S.; Scaccabarozzi D.; Signorini L.; Perego F.; Ilboudo D. P.; Ferrante P.; Delbue S. The use of antimalarial drugs against viral infection. Microorganisms 2020, 8, 85 10.3390/microorganisms8010085. 31936284 PMC7022795

[ref125] Egger M. E.; Huang J. S.; Yin W.; McMasters K. M.; McNally L. R. Inhibition of autophagy with chloroquine is effective in melanoma. J. Surg. Res. 2013, 184, 274–81. 10.1016/j.jss.2013.04.055. 23706562

[ref126] Rashighi M.; Harris J. E. HHS Public Access. Physiol. Behav. 2017, 176, 139–48.28363838

[ref127] Sharma P.; McAlinden K. D.; Ghavami S.; Deshpande D. A. Chloroquine: Autophagy inhibitor, antimalarial, bitter taste receptor agonist in fight against COVID-19, a reality check?. Eur. J. Pharmacol. 2021, 897, 173928 10.1016/j.ejphar.2021.173928. 33545161 PMC7857018

[ref128] Yang Y. P.; Hu L. F.; Zheng H. F.; Mao C. J.; Hu W. D.; Xiong K. P.; Wang F.; Liu C. F. Application and interpretation of current autophagy inhibitors and activators. Acta Pharmacol. Sin. 2013, 34, 625–35. 10.1038/aps.2013.5. 23524572 PMC4002883

[ref129] Manic G.; Obrist F.; Kroemer G.; Vitale I.; Galluzzi L. Chloroquine and hydroxychloroquine for cancer therapy. Mol. Cell. Oncol. 2014, 1, e29911 10.4161/mco.29911. 27308318 PMC4905171

[ref130] Donohue E.; Thomas A.; Maurer N.; Manisali I.; Zeisser-Labouebe M.; Zisman N.; Anderson H. J.; Ng S. S. W.; Webb M.; Bally M.; Roberge M. The Autophagy Inhibitor Verteporfin Moderately Enhances the Antitumor Activity of Gemcitabine in a Pancreatic Ductal Adenocarcinoma Model. J. Cancer 2013, 4, 585–96. 10.7150/jca.7030. 24069069 PMC3781989

[ref131] Barnard R. A.; Wittenburg L. A.; Amaravadi R. K.; Gustafson D. L.; Thorburn A.; Thamm D. H. Phase I clinical trial and pharmacodynamic evaluation of combination hydroxychloroquine and doxorubicin treatment in pet dogs treated for spontaneously occurring lymphoma. Autophagy 2014, 10, 1415–25. 10.4161/auto.29165. 24991836 PMC4203518

[ref132] Zhang J.; et al. Inhibition of hepatocellular stem cells by oncolytic virus targeting Wnt signaling pathway. Prog. Biochem. Biophys. 2017, 44, 326–37. 10.16476/j.pibb.2016.0380.

[ref133] Shanmugam M. K.; Arfuso F.; Kumar A. P.; Wang L.; Goh B. C.; Ahn K. S.; Bishayee A.; Sethi G. Modulation of diverse oncogenic transcription factors by thymoquinone, an essential oil compound isolated from the seeds of Nigella sativa Linn. Pharmacol. Res. 2018, 129, 357–64. 10.1016/j.phrs.2017.11.023. 29162539

[ref134] Racoma I. O.; Meisen W. H.; Wang Q. E.; Kaur B.; Wani A. A. Thymoquinone Inhibits Autophagy and Induces Cathepsin-Mediated, Caspase-Independent Cell Death in Glioblastoma Cells. PLoS One 2013, 8, e72882 10.1371/journal.pone.0072882. 24039814 PMC3767730

[ref135] Efferth T. From ancient herb to modern drug: Artemisia annua and artemisinin for cancer therapy. Semin. Cancer Biol. 2017, 46, 65–83. 10.1016/j.semcancer.2017.02.009. 28254675

[ref136] Ganguli A.; Choudhury D.; Datta S.; Bhattacharya S.; Chakrabarti G. Inhibition of autophagy by chloroquine potentiates synergistically anti-cancer property of artemisinin by promoting ROS dependent apoptosis. Biochimie 2014, 107, 338–49. 10.1016/j.biochi.2014.10.001. 25308836

[ref137] Xu R.; Ji Z.; Xu C.; Zhu J. The clinical value of using chloroquine or hydroxychloroquine as autophagy inhibitors in the treatment of cancers A systematic review and meta-analysis. Med. (United States) 2018, 97, e12912 10.1097/MD.0000000000012912. PMC6257684 30431566

[ref138] Weber S. M.; Chen J.-M.; Levitz S. M. Inhibition of Mitogen-Activated Protein Kinase Signaling by Chloroquine. J. Immunol. 2002, 168, 5303–9. 10.4049/jimmunol.168.10.5303. 11994488

[ref139] Chen M.-Y.; Yadav V. K.; Chu Y. C.; Ong J. R.; Huang T.-Y.; Lee K.-F.; Lee K.-H.; Yeh C.-T.; Lee W.-H. Hydroxychloroquine (HCQ) Modulates Autophagy and Oxidative DNA Damage Stress in Hepatocellular Carcinoma to Overcome Sorafenib Resistance via TLR9/SOD1/hsa-miR-30a-5p/Beclin-1 Axis. Cancers 2021, 13, 3227 10.3390/cancers13133227. 34203465 PMC8267639

[ref140] Wang J.; Qiu L. Drug-induced self-assembled nanovesicles for doxorubicin resistance reversal via autophagy inhibition and delivery synchronism. Theranostics 2022, 12, 3977–94. 10.7150/thno.70852. 35664062 PMC9131275

[ref141] Petiot A.; Ogier-Denis E.; Blommaart E. F. C.; Meijer A. J.; Codogno P. Distinct classes of phosphatidylinositol 3′-kinases are involved in signaling pa7thways that control macroautophagy in HT-29 cells. J. Biol. Chem. 2000, 275, 992–8. 10.1074/jbc.275.2.992. 10625637

[ref142] Mishima Y.; Terui Y.; Mishima Y.; Taniyama A.; Kuniyoshi R.; Takizawa T.; Kimura S.; Ozawa K.; Hatake K. Autophagy and autophagic cell death are next targets for elimination of the resistance to tyrosine kinase inhibitors. Cancer Sci. 2008, 99, 2200–8. 10.1111/j.1349-7006.2008.00932.x. 18823378 PMC11158545

[ref143] Kang R.; Wang Z. H.; Wang B. Q.; Zhang C. M.; Gao W.; Feng Y.; Bai T.; Zhang H. L.; Huang-Pu H.; Wen S. X. Inhibition of autophagy-potentiated chemosensitivity to cisplatin in laryngeal cancer Hep-2 cells. Am. J. Otolaryngol. - Head Neck Med. Surg. 2012, 33, 678–84. 10.1016/j.amjoto.2012.05.005. 22771248

[ref144] Zhao F.; Feng G.; Zhu J.; Su Z.; Guo R.; Liu J.; Zhang H.; Zhai Y. 3-Methyladenine-enhanced susceptibility to sorafenib in hepatocellular carcinoma cells by inhibiting autophagy. Anticancer. Drugs 2021, 32, 386–93. 10.1097/CAD.0000000000001032. 33395067 PMC7952045

[ref145] Tan S.; Peng X.; Peng W.; Zhao Y.; Wei Y. Enhancement of oxaliplatin-induced cell apoptosis and tumor suppression by 3-methyladenine in colon cancer. Oncol. Lett. 2015, 9, 2056–62. 10.3892/ol.2015.2996. 26137012 PMC4467296

[ref146] Tran A. T.; Ramalinga M.; Kedir H.; Clarke R.; Kumar D. Autophagy inhibitor 3-methyladenine potentiates apoptosis induced by dietary tocotrienols in breast cancer cells. Eur. J. Nutr. 2015, 54, 265–72. 10.1007/s00394-014-0707-y. 24830781 PMC4233202

[ref147] Wu F.; Liu Y.; Cheng H.; Meng Y.; Shi J.; Chen Y.; Wu Y. Enhanced cancer starvation therapy based on glucose oxidase/3-methyladenine-loaded dendritic mesoporous organosilicon nanoparticles. Biomolecules 2021, 11, 1363 10.3390/biom11091363. 34572575 PMC8468959

[ref148] Zhang P.; Tang M.; Huang Q.; Zhao G.; Huang N.; Zhang X.; Tan Y.; Cheng Y. Combination of 3-methyladenine therapy and Asn-Gly-Arg (NGR)-modified mesoporous silica nanoparticles loaded with Temozolomide for glioma therapy in vitro. Biochem. Biophys. Res. Commun. 2019, 509, 549–56. 10.1016/j.bbrc.2018.12.158. 30600180

[ref149] Yuan N.; Song L.; Zhang S.; Lin W.; Cao Y.; Xu F.; Fang Y.; Wang Z.; Zhang H.; Li X.; Wang Z.; Cai J.; Wang J.; Zhang Y.; Mao X.; Zhao W.; Hu S.; Chen S.; Wang J. Bafilomycin A1 targets both autophagy and apoptosis pathways in pediatric B-cell acute lymphoblastic leukemia. Haematologica 2015, 100, 345–56. 10.3324/haematol.2014.113324. 25512644 PMC4349273

[ref150] Wu S.; Wang X.; Chen J.; Chen Y. Autophagy of cancer stem cells is involved with chemoresistance of colon cancer cells. Biochem. Biophys. Res. Commun. 2013, 434, 898–903. 10.1016/j.bbrc.2013.04.053. 23624503

[ref151] Greene L. M.; Nolan D. P.; Regan-Komito D.; Campiani G.; Williams D. C.; Zisterer D. M. Inhibition of late-stage autophagy synergistically enhances pyrrolo-1,5-benzoxazepine-6-induced apoptotic cell death in human colon cancer cells. Int. J. Oncol. 2013, 43, 927–35. 10.3892/ijo.2013.1989. 23799546

[ref152] Michel V.; Licon-Munoz Y.; Trujillo K.; Bisoffi M.; Parra K. J. Inhibitors of vacuolar ATPase proton pumps inhibit human prostate cancer cell invasion and prostate-specific antigen expression and secretion. Int. J. Cancer 2013, 132, E1–E10. 10.1002/ijc.27811. 22945374 PMC3504192

[ref153] Ünal T. D.; Hamurcu Z.; Delibaşı N.; Çınar V.; Güler A.; Gökçe S.; Nurdinov N.; Ozpolat B. Thymoquinone Inhibits Proliferation and Migration of MDA-MB-231 Triple Negative Breast Cancer Cells by Suppressing Autophagy, Beclin-1 and LC3. Anticancer. Agents Med. Chem. 2021, 21, 355–64. 10.2174/1871520620666200807221047. 32767958

[ref154] Chu H. Y.; Wang W.; Chen X.; Jiang Y. E.; Cheng R.; Qi X.; Zhong Z. M.; Zeng M. S.; Zhu X. F.; Sun C. Z. Bafilomycin A1 increases the sensitivity of tongue squamous cell carcinoma cells to cisplatin by inhibiting the lysosomal uptake of platinum ions but not autophagy. Cancer Lett. 2018, 423, 105–12. 10.1016/j.canlet.2018.03.003. 29524554

[ref155] Ma K.; Li S.; Huo X.; Guo M.; Du X.; Li C.; Liu X.; Lv J.; Chen Z. Exploring the mechanism of cisplatin resistance by transcriptome sequencing and reversing the chemoresistance by autophagy inhibition in small cell lung cancer. Biochem. Biophys. Res. Commun. 2020, 533, 474–80. 10.1016/j.bbrc.2020.09.023. 32977950

[ref156] Yan Y.; Jiang K.; Liu P.; Zhang X.; Dong X.; Gao J.; Liu Q.; Barr M. P.; Zhang Q.; Hou X.; Meng S.; Gong P. Bafilomycin A1 induces caspase-independent cell death in hepatocellular carcinoma cells via targeting of autophagy and MAPK pathways. Sci. Rep. 2016, 6, 37052 10.1038/srep37052. 27845389 PMC5109251

[ref157] Guo W.; Wang Y.; Wang Z.; Wang Y. P.; Zheng H. Inhibiting autophagy increases epirubicin’s cytotoxicity in breast cancer cells. Cancer Sci. 2016, 107, 1610–21. 10.1111/cas.13059. 27560771 PMC5132286

[ref158] Lin J. F.; Lin Y. C.; Yang S. C.; Tsai T. F.; Chen H. E.; Chou K. Y.; Hwang T. I. S. Autophagy inhibition enhances RAD001-induced cytotoxicity in human bladder cancer cells. Drug Des. Devel. Ther. 2016, 10, 1501–13. 10.2147/DDDT.S95900. PMC4841413 27143856

[ref159] Bosnjak M.; Ristic B.; Arsikin K.; Mircic A.; Suzin-Zivkovic V.; Perovic V.; Bogdanovic A.; Paunovic V.; Markovic I.; Bumbasirevic V.; Trajkovic V.; Harhaji-Trajkovic L. Inhibition of mTOR-dependent autophagy sensitizes leukemic cells to cytarabine-induced apoptotic death. PLoS One 2014, 9, e94374 10.1371/journal.pone.0094374. 24714637 PMC3979773

[ref160] Ahmadzada T.; Reid G.; McKenzie D. R. Fundamentals of siRNA and miRNA therapeutics and a review of targeted nanoparticle delivery systems in breast cancer. Biophys. Rev. 2018, 10, 69–86. 10.1007/s12551-017-0392-1. 29327101 PMC5803180

[ref161] Liu H.; Chen Z.; Jin W.; Barve A.; Wan Y. J. Y.; Cheng K. Silencing of α-complex protein-2 reverses alcohol- and cytokine-induced fibrogenesis in hepatic stellate cells. Liver Res. 2017, 1, 70–9. 10.1016/j.livres.2017.05.003. 28966795 PMC5613955

[ref162] Carthew R. W.; Sontheimer E. J. Origins and Mechanisms of miRNAs and siRNAs. Cell 2009, 136, 642–55. 10.1016/j.cell.2009.01.035. 19239886 PMC2675692

[ref163] Huang T.; Kim C. K.; Alvarez A. A.; Pangeni R. P.; Wan X.; Song X.; Shi T.; Yang Y.; Sastry N.; Horbinski C. M.; Lu S.; Stupp R.; Kessler J. A.; Nishikawa R.; Nakano I.; Sulman E. P.; Lu X.; James C. D.; Yin X. M.; Hu B.; Cheng S. Y. MST4 Phosphorylation of ATG4B Regulates Autophagic Activity, Tumorigenicity, and Radioresistance in Glioblastoma. Cancer Cell 2017, 32, 840–855. 10.1016/j.ccell.2017.11.005. 29232556 PMC5734934

[ref164] Frixa T.; Donzelli S.; Blandino G. Oncogenic MicroRNAs: Key players in malignant transformation. Cancers (Basel). 2015, 7, 2466–85. 10.3390/cancers7040904. 26694467 PMC4695904

[ref165] Peng Y.; Croce C. M. The role of microRNAs in human cancer. Signal Transduct. Target. Ther. 2016, 1, 15004 10.1038/sigtrans.2015.4. 29263891 PMC5661652

[ref166] Arlotta P.; Macklis J. D. Archeo-cell biology: Carbon dating is not just for pots and dinosaurs. Cell 2005, 122, 4–6. 10.1016/j.cell.2005.06.037. 16009125

[ref167] Yin P. T.; Shah B. P.; Lee K. B. Combined magnetic nanoparticle-based MicroRNA and hyperthermia therapy to enhance apoptosis in brain cancer cells. Small 2014, 10, 4106–12. 10.1002/smll.201400963. 24947843 PMC4206574

[ref168] Jiang L. H.; Zhang H Da; Tang J. H. MiR-30a: A Novel Biomarker and Potential Therapeutic Target for Cancer. J. Oncol. 2018, 2018, 15167829 10.1155/2018/5167829. PMC6106977 30158978

[ref169] Santulli G. microRNA: Cancer. From Molecular Biology to Clinical Practice, Advances in Experimental Medicine and Biology 889; Springer, 2015; p 36.

[ref170] Tili E.; Michaille J. J.; Croce C. M. MicroRNAs play a central role in molecular dysfunctions linking inflammation with cancer. Immunol. Rev. 2013, 253, 167–84. 10.1111/imr.12050. 23550646

[ref171] Rupaimoole R.; Calin G. A.; Lopez-Berestein G.; Sood A. K. MiRNA deregulation in cancer cells and the tumor microenvironment. Cancer Discovery 2016, 6, 235–46. 10.1158/2159-8290.CD-15-0893. 26865249 PMC4783232

[ref172] Acunzo M.; Romano G.; Wernicke D.; Croce C. M. MicroRNA and cancer - A brief overview. Adv. Biol. Regul. 2015, 57, 1–9. 10.1016/j.jbior.2014.09.013. 25294678

[ref173] Bonci D.; Coppola V.; Musumeci M.; Addario A.; Giuffrida R.; Memeo L.; D’Urso L.; Pagliuca A.; Biffoni M.; Labbaye C.; Bartucci M.; Muto G.; Peschle C.; De Maria R. The miR-15a-miR-16–1 cluster controls prostate cancer by targeting multiple oncogenic activities. Nat. Med. 2008, 14, 1271–7. 10.1038/nm.1880. 18931683

[ref174] Kota J.; Chivukula R. R.; O’Donnell K. A.; Wentzel E. A.; Montgomery C. L.; Hwang H. W.; Chang T. C.; Vivekanandan P.; Torbenson M.; Clark K. R.; Mendell J. R.; Mendell J. T. Therapeutic microRNA Delivery Suppresses Tumorigenesis in a Murine Liver Cancer Model. Cell 2009, 137, 1005–17. 10.1016/j.cell.2009.04.021. 19524505 PMC2722880

[ref175] Kinsey C. G.; Camolotto S. A.; Boespflug A. M.; Guillen K. P.; Foth M.; Truong A.; Schuman S. S.; Shea J. E.; Seipp M. T.; Yap J. T.; Burrell L. D.; Lum D. H.; Whisenant J. R.; Gilcrease G. W.; Cavalieri C. C.; Rehbein K. M.; Cutler S. L.; Affolter K. E.; Welm A. L.; Welm B. E.; Scaife C. L.; Snyder E. L.; McMahon M. Protective autophagy elicited by RAF→MEK→ERK inhibition suggests a treatment strategy for RAS-driven cancers. Nat. Med. 2019, 25, 620–7. 10.1038/s41591-019-0367-9. 30833748 PMC6452642

[ref176] Zhu H.; Wu H.; Liu X.; Li B.; Chen Y.; Ren X.; Liu C. G.; Yang J. M. Regulation of autophagy by a beclin 1-targeted microRNA, miR-30a, in cancer cells. Autophagy 2009, 5, 816–23. 10.4161/auto.9064. 19535919 PMC3669137

[ref177] Zou Z.; Wu L.; Ding H.; Wang Y.; Zhang Y.; Chen X.; Chen X.; Zhang C. Y.; Zhang Q.; Zen K. MicroRNA-30a sensitizes tumor cells to cis-platinum via suppressing beclin 1-mediated autophagy. J. Biol. Chem. 2012, 287, 4148–56. 10.1074/jbc.M111.307405. 22157765 PMC3281695

[ref178] Yu Y.; Cao L.; Yang L.; Kang R.; Lotze M.; Tang D. microRNA 30A promotes autophagy in response to cancer therapy. Autophagy 2012, 8, 853–5. 10.4161/auto.20053. 22617440 PMC3378424

[ref179] Huang Y.; Chuang A. Y.; Ratovitski E. A. Phospho-ΔNp63α/miR-885–3p axis in tumor cell life and cell death upon cisplatin exposure. Cell Cycle 2011, 10, 3938–47. 10.4161/cc.10.22.18107. 22071691 PMC3266119

[ref180] Cai Y.; An B.; Yao D.; Zhou H.; Zhu J. MicroRNA miR-30a inhibits cisplatin resistance in ovarian cancer cells through autophagy. Bioengineered 2021, 12, 10713–22. 10.1080/21655979.2021.2001989. 34747309 PMC8810079

[ref181] Lin X.; Lai X.; Feng W.; Yu X.; Gu Q.; Zheng X. MiR-30a sensitized lung cancer against neoadjuvant chemotherapy by depressing autophagy. Jpn. J. Clin. Oncol. 2021, 51, 675–84. 10.1093/jjco/hyaa272. 33537721

[ref182] Xu C.-G.; Yang M.-F.; Fan J.-X.; Wang W. MiR-30a and miR-205 are downregulated in hypoxia and modulate radiosensitivity of prostate cancer cells by inhibiting autophagy via TP53INP1. Eur. Rev. Med. Pharmacol. Sci. 2016, 20, 1501–8. 27160121

[ref183] Yang J.; Rao S.; Cao R.; Xiao S.; Cui X.; Ye L. miR-30a-5p suppresses lung squamous cell carcinoma via ATG5 -mediated autophagy. Aging (Albany. NY). 2021, 13, 17462–72. 10.18632/aging.203235. 34253689 PMC8312466

[ref184] Varambally S.; Cao Q.; Mani R.-S.; Shankar S.; Wang X.; Ateeq B.; Laxman B.; Cao X.; Jing X.; Ramnarayanan K.; et al. Genomic Loss of microRNA-101 Leads to Overexpression of Histone Methyltransferase EZH2 in Cancer. Science 2008, 322, 1695–9. 10.1126/science.1165395. 19008416 PMC2684823

[ref185] Li J. T.; Jia L. T.; Liu N. N.; Zhu X. S.; Liu Q. Q.; Wang X. L.; Yu F.; Liu Y. L.; Yang A. G.; Gao C. F. MiRNA-101 inhibits breast cancer growth and metastasis by targeting CX chemokine receptor7. Oncotarget 2015, 6, 30818–30. 10.18632/oncotarget.5067. 26360780 PMC4741570

[ref186] Zhang J. G.; Guo J. F.; Liu D. L.; Liu Q.; Wang J. J. MicroRNA-101 exerts tumor-suppressive functions in non-small cell lung cancer through directly targeting enhancer of zeste homolog 2. J. Thorac. Oncol. 2011, 6, 671–8. 10.1097/JTO.0b013e318208eb35. 21270667

[ref187] Jing Z.; Han W.; Sui X.; Xie J.; Pan H. Interaction of autophagy with microRNAs and their potential therapeutic implications in human cancers. Cancer Lett. 2015, 356, 332–8. 10.1016/j.canlet.2014.09.039. 25304373

[ref188] Frankel L. B.; Wen J.; Lees M.; Høyer-Hansen M.; Farkas T.; Krogh A.; Jäättelä M.; Lund A. H. MicroRNA-101 is a potent inhibitor of autophagy. EMBO J. 2011, 30, 4628–41. 10.1038/emboj.2011.331. 21915098 PMC3243595

[ref189] Xu L.; Beckebaum S.; Iacob S.; Wu G.; Kaiser G. M.; Radtke A.; Liu C.; Kabar I.; Schmidt H. H.; Zhang X.; Lu M.; Cicinnati V. R. MicroRNA-101 inhibits human hepatocellular carcinoma progression through EZH2 downregulation and increased cytostatic drug sensitivity. J. Hepatol. 2014, 60, 590–8. 10.1016/j.jhep.2013.10.028. 24211739

[ref190] Xu Y.; An Y.; Wang Y.; Zhang C.; Zhang H.; Huang C.; Jiang H.; Wang X.; Li X. miR-101 inhibits autophagy and enhances cisplatin-induced apoptosis in hepatocellular carcinoma cells. Oncol. Rep. 2013, 29, 2019–24. 10.3892/or.2013.2338. 23483142

[ref191] Zachari M.; Ganley I. G. The mammalian ULK1 complex and autophagy initiation. Essays Biochem. 2017, 61, 585–96. 10.1042/EBC20170021. 29233870 PMC5869855

[ref192] Tang F.; Hu P.; Yang Z.; Xue C.; Gong J.; Sun S.; Shi L.; Zhang S.; Li Z.; Yang C.; Zhang J.; Xie C. SBI0206965, a novel inhibitor of Ulk1, suppresses non-small cell lung cancer cell growth by modulating both autophagy and apoptosis pathways. Oncol. Rep. 2017, 37, 3449–58. 10.3892/or.2017.5635. 28498429

[ref193] Dower C. M.; Bhat N.; Gebru M. T.; Chen L.; Wills C. A.; Miller B. A.; Wang H. G. Targeted inhibition of ULK1 promotes apoptosis and suppresses tumor growth and metastasis in neuroblastoma. Mol. Cancer Ther. 2018, 17, 2365–76. 10.1158/1535-7163.MCT-18-0176. 30166400 PMC6215526

[ref194] Chen H.; Zhang Z.; Lu Y.; Song K.; Liu X.; Xia F.; Sun W. Downregulation of ULK1 by microRNA-372 inhibits the survival of human pancreatic adenocarcinoma cells. Cancer Sci. 2017, 108, 1811–9. 10.1111/cas.13315. 28677209 PMC5581518

[ref195] Huang L.; Hu C.; Li H.; Cao H.; Wu X.; Wang R.; Lu H.; Chen H. MicroRNA-29c Increases the Chemosensitivity of Pancreatic Cancer Cells by Inhibiting USP22 Mediated Autophagy. Cell. Physiol. Biochem. 2018, 47, 747–58. 10.1159/000490027. 29807360

[ref196] Kwon J. J.; Willy J. A.; Quirin K. A.; Wek R. C.; Korc M.; Yin X. M.; Kota J. Novel role of miR-29a in pancreatic cancer autophagy and its therapeutic potential. Oncotarget 2016, 7, 71635–50. 10.18632/oncotarget.11928. 27626694 PMC5342107

[ref197] Suren D.; Yildirim M.; Alikanoglu A. S.; Kaya V.; Yildiz M.; Dilli U. D.; Sezer C. The role of High Mobility Group Box 1 (HMGB1) in colorectal cancer. Med. Sci. Monit. 2014, 20, 530–7. 10.12659/MSM.890531. 24681824 PMC3976146

[ref198] Cheng Y.; Wang H.; Mao M.; Liang C.; Zhang Y.; Yang D.; Wei Z.; Gao S.; Hu B.; Wang L.; Cai Q. Escin Increases the Survival Rate of LPS-Induced Septic Mice Through Inhibition of HMGB1 Release from Macrophages. Cell. Physiol. Biochem. 2015, 36, 1577–86. 10.1159/000430320. 26159678

[ref199] Xiong J.; Wang D.; Wei A.; Ke N.; Wang Y.; Tang J.; He S.; Hu W.; Liu X. MicroRNA-410–3p attenuates gemcitabine resistance in pancreatic ductal adenocarcinoma by inhibiting HMGB1-mediated autophagy. Oncotarget 2017, 8, 107500–12. 10.18632/oncotarget.22494. 29296182 PMC5746084

[ref200] Liu L.; Ren W.; Chen K. MiR-34a promotes apoptosis and inhibits autophagy by targeting HMGB1 in acute myeloid leukemia cells. Cell. Physiol. Biochem. 2017, 41, 1981–92. 10.1159/000475277. 28478444

[ref201] Chang E.; Eddins-Folensbee F.; Coverdale J. Survey of the Prevalence of Burnout, Stress, Depression, and the Use of Supports by Medical Students at One School. Acad. Psychiatry 2012, 36, 177–82. 10.1176/appi.ap.11040079. 22751817

[ref202] Minister of Health Regulation Number 43 of 2019 Concerning Community Health Centers, Ministry of Health, Jakarta, Indonesia, 2019

[ref203] Yu M.-M.; Wang G.-j.; Wu K.-H.; Xue S.-L.; Ju L.- L.; Li Q.-r.; Xiong A.-W.; Yin G.-p. MicroRNA-373–3p inhibits the growth of cervical cancer by targeting AKT1 both in vitro and in vivo. Acta Biochim. Polym. 2021, 68, 611–7. 10.18388/abp.2020_5446. 34236826

[ref204] Lu R.; Yang Z.; Xu G.; Yu S. miR-338 modulates proliferation and autophagy by PI3K/AKT/mTOR signaling pathway in cervical cancer. Biomed. Pharmacother. 2018, 105, 633–44. 10.1016/j.biopha.2018.06.024. 29898430

[ref205] Wei F.; Wang Q.; Su Q.; Huang H.; Luan J.; Xu X.; Wang J. miR-373 Inhibits Glioma Cell U251 Migration and Invasion by Down-Regulating CD44 and TGFBR2. Cell. Mol. Neurobiol. 2016, 36, 1389–97. 10.1007/s10571-016-0338-3. 26858153 PMC11482453

[ref206] Menghini R.; Casagrande V.; Marino A.; Marchetti V.; Cardellini M.; Stoehr R.; Rizza S.; Martelli E.; Greco S.; Mauriello A.; Ippoliti A.; Martelli F.; Lauro R.; Federici M. MiR-216a: A link between endothelial dysfunction and autophagy. Cell Death Dis. 2014, 5, e1029 10.1038/cddis.2013.556. 24481443 PMC4040670

[ref207] Pennati M.; Lopergolo A.; Profumo V.; De Cesare M.; Sbarra S.; Valdagni R.; Zaffaroni N.; Gandellini P.; Folini M. miR-205 impairs the autophagic flux and enhances cisplatin cytotoxicity in castration-resistant prostate cancer cells. Biochem. Pharmacol. 2014, 87, 579–97. 10.1016/j.bcp.2013.12.009. 24370341

[ref208] Wang Z.; Ting Z.; Li Y.; Chen G.; Lu Y.; Hao X. microRNA-199a is able to reverse cisplatin resistance in human ovarian cancer cells through the inhibition of mammalian target of rapamycin. Oncol Lett. 2013, 6, 789–94. 10.3892/ol.2013.1448. 24137412 PMC3789061

[ref209] Weng Y.; Xiao H.; Zhang J.; Liang X. J.; Huang Y. RNAi therapeutic and its innovative biotechnological evolution. Biotechnol. Adv. 2019, 37, 801–25. 10.1016/j.biotechadv.2019.04.012. 31034960

[ref210] Agarwal S.; Simon A. R.; Goel V.; Habtemariam B. A.; Clausen V. A.; Kim J. B.; Robbie G. J. Pharmacokinetics and Pharmacodynamics of the Small Interfering Ribonucleic Acid, Givosiran, in Patients With Acute Hepatic Porphyria. Clin. Pharmacol. Ther. 2020, 108, 63–72. 10.1002/cpt.1802. 31994716

[ref211] Apel A.; Herr I.; Schwarz H.; Rodemann H. P.; Mayer A. Blocked autophagy sensitizes resistant carcinoma cells to radiation therapy. Cancer Res. 2008, 68, 1485–94. 10.1158/0008-5472.CAN-07-0562. 18316613

[ref212] Daniel F.; Legrand A.; Pessayre D.; Vadrot N.; Descatoire V.; Bernuau D. Partial Beclin 1 silencing aggravates doxorubicin- and Fas-induced apoptosis in HepG2 cells. World J. Gastroenterol. 2006, 12, 2895–900. 10.3748/wjg.v12.i18.2895. 16718815 PMC4087807

[ref213] Han W.; Sun J.; Feng L.; Wang K. F.; Li D.; Pan Q.; Chen Y.; Jin W.; Wang X.; Pan H.; Jin H. Autophagy inhibition enhances daunorubicin-induced apoptosis in K562 cells. PLoS One 2011, 6, e28491 10.1371/journal.pone.0028491. 22164300 PMC3229606

[ref214] Shimizu S.; Takehara T.; Hikita H.; Kodama T.; Tsunematsu H.; Miyagi T.; Hosui A.; Ishida H.; Tatsumi T.; Kanto T.; Hiramatsu N.; Fujita N.; Yoshimori T.; Hayashi N. Inhibition of autophagy potentiates the antitumor effect of the multikinase inhibitor sorafenib in hepatocellular carcinoma. Int. J. Cancer 2012, 131, 548–57. 10.1002/ijc.26374. 21858812

[ref215] Zheng B.; Zhu H.; Gu D.; Pan X.; Qian L.; Xue B.; Yang D.; Zhou J.; Shan Y. MiRNA-30a-mediated autophagy inhibition sensitizes renal cell carcinoma cells to sorafenib. Biochem. Biophys. Res. Commun. 2015, 459, 234–9. 10.1016/j.bbrc.2015.02.084. 25712526

[ref216] Ikeda A. K.; Judelson D. R.; Federman N.; Glaser K. B.; Landaw E. M.; Denny C. T.; Sakamoto K. M. ABT-869 inhibits the proliferation of ewing sarcoma cells and suppresses platelet-derived growth factor receptor β and c-KIT signaling pathways. Mol. Cancer Ther. 2010, 9, 653–60. 10.1158/1535-7163.MCT-09-0812. 20197394 PMC2837519

[ref217] Pan H.; Wang Z.; Jiang L.; Sui X.; You L.; Shou J.; Jing Z.; Xie J.; Ge W.; Cai X.; Huang W.; Han W. Autophagy inhibition sensitizes hepatocellular carcinoma to the multikinase inhibitor linifanib. Sci. Rep. 2014, 4, 6683 10.1038/srep06683. 25327881 PMC4202209

[ref218] Noman M. Z.; Janji B.; Kaminska B.; Van Moer K.; Pierson S.; Przanowski P.; Buart S.; Berchem G.; Romero P.; Mami-Chouaib F.; Chouaib S. Blocking hypoxia-induced autophagy in tumors restores cytotoxic T-cell activity and promotes regression. Cancer Res. 2011, 71, 5976–86. 10.1158/0008-5472.CAN-11-1094. 21810913

[ref219] Yang L.; Yang M.; Zhang H.; Wang Z.; Yu Y.; Xie M.; Zhao M.; Liu L.; Cao L. S100A8-targeting siRNA enhances arsenic trioxide-induced myeloid leukemia cell death by down-regulating autophagy. Int. J. Mol. Med. 2012, 29, 65–72. 10.3892/ijmm.2011.806. 21971985

[ref220] Peng X.; Gong F.; Chen Y.; Jiang Y.; Liu J.; Yu M.; Zhang S.; Wang M.; Xiao G.; Liao H. Autophagy promotes paclitaxel resistance of cervical cancer cells: Involvement of Warburg effect activated hypoxia-induced factor 1-α-mediated signaling. Cell Death Dis. 2014, 5, e1367 10.1038/cddis.2014.297. 25118927 PMC4454295

[ref221] Xiong X.; Wu M.; Zhang H.; Li J.; Lu B.; Guo Y.; Zhou T.; Guo H.; Peng R.; Li X.; Tian Q.; Wang Y. Atg5 siRNA inhibits autophagy and enhances norcantharidin-induced apoptosis in hepatocellular carcinoma. Int. J. Oncol. 2015, 47, 1321–8. 10.3892/ijo.2015.3103. 26240015

[ref222] Wang Y.; Jiang W.; Li C.; Xiong X.; Guo H.; Tian Q.; Li X. Autophagy Suppression Accelerates Apoptosis Induced by Norcantharidin in Cholangiocarcinoma. Pathol. Oncol. Res. 2020, 26, 1697–707. 10.1007/s12253-019-00719-9. 31612378

[ref223] Zanotto-Filho A.; Braganhol E.; Klafke K.; Figueiró F.; Terra S. R.; Paludo F. J.; Morrone M.; Bristot I. J.; Battastini A. M.; Forcelini C. M.; Bishop A. J. R.; Gelain D. P.; Moreira J. C. F. Autophagy inhibition improves the efficacy of curcumin/Temozolomide combination therapy in glioblastomas. Cancer Lett. 2015, 358, 220–31. 10.1016/j.canlet.2014.12.044. 25542083

[ref224] Wang J.; Qi Q.; Zhou W.; Feng Z.; Huang B.; Chen A.; Zhang D.; Li W.; Zhang Q.; Jiang Z.; Bjerkvig R.; Prestegarden L.; Thorsen F.; Wang X.; Li X.; Wang J. Inhibition of glioma growth by flavokawain B is mediated through endoplasmic reticulum stress induced autophagy. Autophagy 2018, 14, 2007–22. 10.1080/15548627.2018.1501133. 30025493 PMC6152528

[ref225] Quan Y.; Lei H.; Wahafu W.; Liu Y.; Ping H.; Zhang X. Inhibition of autophagy enhances the anticancer effect of enzalutamide on bladder cancer. Biomed. Pharmacother. 2019, 120, 109490 10.1016/j.biopha.2019.109490. 31574376

[ref226] Liu W.; Lo Y. L.; Hsu C.; Wu Y. T.; Liao Z. X.; Wu W. J.; Chen Y. J.; Kao C.; Chiu C. C.; Wang L. F. CS-PEI/Beclin-siRNA Downregulate Multidrug Resistance Proteins and Increase Paclitaxel Therapeutic Efficacy against NSCLC. Mol. Ther. - Nucleic Acids 2019, 17, 477–90. 10.1016/j.omtn.2019.06.017. 31336235 PMC6656922

[ref227] Dréno B.; Kunstfeld R.; Hauschild A.; Fosko S.; Zloty D.; Labeille B.; Grob J. J.; Puig S.; Gilberg F.; Bergström D.; Page D. R.; Rogers G.; Schadendorf D. Two intermittent vismodegib dosing regimens in patients with multiple basal-cell carcinomas (MIKIE): a randomised, regimen-controlled, double-blind, phase 2 trial. Lancet Oncol. 2017, 18, 404–12. 10.1016/S1470-2045(17)30072-4. 28188086

[ref228] Fan J.; Zhang X.; Wang S.; Chen W.; Li Y.; Zeng X.; Wang Y.; Luan J.; Li L.; Wang Z.; Sun X.; Shen B.; Ju D. Regulating autophagy facilitated therapeutic efficacy of the sonic Hedgehog pathway inhibition on lung adenocarcinoma through GLI2 suppression and ROS production. Cell Death Dis. 2019, 10, 626 10.1038/s41419-019-1840-6. 31427566 PMC6700102

[ref229] Zheng W.; Chen Q.; Wang C.; Yao D.; Zhu L.; Pan Y.; Zhang J.; Bai Y.; Shao C. Inhibition of Cathepsin D (CTSD) enhances radiosensitivity of glioblastoma cells by attenuating autophagy. Mol. Carcinog. 2020, 59, 651–60. 10.1002/mc.23194. 32253787

[ref400] Gokcek O. C. LC3 siRNA-Mediated Impressed Autophagy in GBM Cells Enhances the Efficacy of Temozolomide: Inhibits Proliferation, Clone Formation and Migration of U87-MG Cells. Academic Journal of Health Sciences 2024, 39 (3), 121–128.

[ref230] Zhang X.; Wang L. L.; Wang B.; Liu H. L.; Zhang J.; Li Y. H.; Wang L. H. Effect of siRNA-induced Atg7 gene silencing on the sensitivity of ovarian cancer SKOV3 cells to cisplatin. Am. J. Transl. Res. 2020, 12, 2052–61. 32509199 PMC7269981

[ref231] Deng S.; Shanmugam M. K.; Kumar A. P.; Yap C. T.; Sethi G.; Bishayee A. Targeting autophagy using natural compounds for cancer prevention and therapy. Cancer 2019, 125, 1228–46. 10.1002/cncr.31978. 30748003

[ref401] Tunc C. U.; Aydin O. Co-delivery of Bcl-2 siRNA and doxorubicin through gold nanoparticle-based delivery system for a combined cancer therapy approach. Journal of Drug Delivery Science and Technology 2022, 74, 103603 10.1016/j.jddst.2022.103603.

[ref232] Wang J.; Yu Y.; Lu K.; Yang M.; Li Y.; Zhou X.; Sun Z. Silica nanoparticles induce autophagy dysfunction via lysosomal impairment and inhibition of autophagosome degradation in hepatocytes. Int. J. Nanomedicine 2017, 12, 809–25. 10.2147/IJN.S123596. 28182147 PMC5279829

[ref233] Yoon M. S. Nanotechnology-based targeting of mTOR signaling in cancer. Int. J. Nanomedicine 2020, 15, 5767–81. 10.2147/IJN.S254574. 32821100 PMC7418174

[ref234] Mokhtarieh A. A.; Cheong S.; Kim S.; Chung B. H.; Lee M. K. Asymmetric liposome particles with highly efficient encapsulation of siRNA and without nonspecific cell penetration suitable for target-specific delivery. Biochim. Biophys. Acta - Biomembr. 2012, 1818, 1633–41. 10.1016/j.bbamem.2012.03.016. 22465072

[ref235] Zhang D.; Lv P.; Zhou C.; Zhao Y.; Liao X.; Yang B. Cyclodextrin-based delivery systems for cancer treatment. Mater. Sci. Eng., C 2019, 96, 872–86. 10.1016/j.msec.2018.11.031. 30606602

[ref236] Junghanns J. U. A. H.; Müller R. H. Nanocrystal technology, drug delivery and clinical applications. Int. J. Nanomedicine 2008, 3, 295–309. 10.2147/IJN.S595. 18990939 PMC2626933

[ref237] Colombo M.; Staufenbiel S.; Rühl E.; Bodmeier R. In situ determination of the saturation solubility of nanocrystals of poorly soluble drugs for dermal application. Int. J. Pharm. 2017, 521, 156–66. 10.1016/j.ijpharm.2017.02.030. 28223247

[ref238] Ganta S.; Paxton J. W.; Baguley B. C.; Garg S. Formulation and pharmacokinetic evaluation of an asulacrine nanocrystalline suspension for intravenous delivery. Int. J. Pharm. 2009, 367, 179–86. 10.1016/j.ijpharm.2008.09.022. 18848873

[ref239] Wang T.; Qi J.; Ding N.; Dong X.; Zhao W.; Lu Y.; Wang C.; Wu W. Tracking translocation of self-discriminating curcumin hybrid nanocrystals following intravenous delivery. Int. J. Pharm. 2018, 546, 10–9. 10.1016/j.ijpharm.2018.05.020. 29751141

[ref240] Xin X.; Du X.; Xiao Q.; Azevedo H. S.; He W.; Yin L. Drug Nanorod-Mediated Intracellular Delivery of microRNA-101 for Self-sensitization via Autophagy Inhibition. Nano-Micro Lett. 2019, 11, 82 10.1007/s40820-019-0310-0. PMC7770860 34138035

[ref241] Liechty W. B.; Kryscio D. R.; Slaughter B. V.; Peppas N. A. Polymers for drug delivery systems. Annu. Rev. Chem. Biomol. Eng. 2010, 1, 149–73. 10.1146/annurev-chembioeng-073009-100847. 22432577 PMC3438887

[ref242] Jia H. Z.; Zhang W.; Zhu J. Y.; Yang B.; Chen S.; Chen G.; Zhao Y. F.; Feng J.; Zhang X. Z. Hyperbranched-hyperbranched polymeric nanoassembly to mediate controllable co-delivery of siRNA and drug for synergistic tumor therapy. J. Controlled Release 2015, 216, 9–17. 10.1016/j.jconrel.2015.08.006. 26272764

[ref243] Li H.; Sun Y.; Liang J.; Fan Y.; Zhang X. PH-Sensitive pullulan-DOX conjugate nanoparticles for co-loading PDTC to suppress growth and chemoresistance of hepatocellular carcinoma. J. Mater. Chem. B 2015, 3, 8070–8. 10.1039/C5TB01210D. 32262864

[ref244] Chen L.; Qian M.; Zhang L.; Xia J.; Bao Y.; Wang J.; Guo L.; Li Y. Co-delivery of doxorubicin and shRNA of Beclin1 by folate receptor targeted pullulan-based multifunctional nanomicelles for combinational cancer therapy. RSC Adv. 2018, 8, 17710–22. 10.1039/C8RA01679H. 35542072 PMC9080481

[ref245] Song Z.; Liang X.; Wang Y.; Han H.; Yang J.; Fang X.; Li Q. Phenylboronic acid-functionalized polyamidoamine-mediated miR-34a delivery for the treatment of gastric cancer. Biomater. Sci. 2019, 7, 1632–42. 10.1039/C8BM01385C. 30720809

[ref246] Wang H.; Zhao X.; Guo C.; Ren D.; Zhao Y.; Xiao W.; Jiao W. Aptamer-Dendrimer Bioconjugates for Targeted Delivery of miR-34a Expressing Plasmid and Antitumor Effects in Non-Small Cell Lung Cancer Cells. PLoS One 2015, 10, e0139136 10.1371/journal.pone.0139136. 26406332 PMC4583438

[ref247] Meng H.; Liong M.; Xia T.; Li Z.; Ji Z.; Zink J. I.; Nel A. E. Engineered design of mesoporous silica nanoparticles to deliver doxorubicin and p-glycoprotein siRNA to overcome drug resistance in a cancer cell line. ACS Nano 2010, 4, 4539–50. 10.1021/nn100690m. 20731437 PMC3899722

[ref248] Han H.; Chen W.; Yang J.; Zhang J.; Li Q.; Yang Y. 2-Amino-6-chloropurine-modified polyamidoamine-mediated p53 gene transfection to achieve anti-tumor efficacy. New J. Chem. 2018, 42, 13375–81. 10.1039/C8NJ01870G.

[ref249] Wang C.; Han M.; Liu X.; Chen S.; Hu F.; Sun J.; Yuan H. Mitoxantrone-preloaded water-responsive phospholipid-amorphous calcium carbonate hybrid nanoparticles for targeted and effective cancer therapy. Int. J. Nanomedicine 2019, 14, 1503–17. 10.2147/IJN.S193976. 30880961 PMC6396884

[ref250] Wang C.; Li M.; Yang T.; Ding X.; Bao X.; Ding Y.; Xiong H.; Wu Y.; Wang W.; Zhou J. A self-assembled system for tumor-targeted co-delivery of drug and gene. Mater. Sci. Eng., C 2015, 56, 280–5. 10.1016/j.msec.2015.06.034. 26249591

[ref251] Yang B.; Hao A.; Chen L. Mirror siRNAs loading for dual delivery of doxorubicin and autophagy regulation siRNA for multidrug reversing chemotherapy. Biomed. Pharmacother. 2020, 130, 110490 10.1016/j.biopha.2020.110490. 32712530

[ref252] Nagpal K.; Singh S. K.; Mishra D. N. Chitosan nanoparticles: A promising system in novel drug delivery. Chem. Pharm. Bull. 2010, 58, 1423–30. 10.1248/cpb.58.1423. 21048331

[ref253] Bhumkar R. D.; Pokharkar V. B. Studies on effect of pH on cross-linking of Chitosan with sodium tripolyphosphate: A technical note. AAPS PharmSciTech 2006, 7, E138 10.1208/pt070250. 16796367 PMC2750277

[ref254] Zheng Y.; Su C.; Zhao L.; Shi Y. Chitosan nanoparticle-mediated co-delivery of shAtg-5 and gefitinib synergistically promoted the efficacy of chemotherapeutics through the modulation of autophagy. J. Nanobiotechnology 2017, 15, 28 10.1186/s12951-017-0261-x. 28399862 PMC5387274

[ref255] Gulzar A.; Yang P.; He F.; Xu J.; Yang D.; Xu L.; Jan M. O. Bioapplications of graphene constructed functional nanomaterials. Chem. Biol. Interact. 2017, 262, 69–89. 10.1016/j.cbi.2016.11.019. 27876601

[ref256] Nie W.; Xu M. D.; Gan L.; Huang H.; Xiu Q.; Li B. Overexpression of stathmin 1 is a poor prognostic biomarker in non-small cell lung cancer. Lab. Investig. 2015, 95, 56–64. 10.1038/labinvest.2014.124. 25384122

[ref257] Hsieh S.-Y.; Huang S.-F.; Yu M.-C.; Yeh T.-S.; Chen T.-C.; Lin Y.-J.; Chang C.-J.; Sung C.-M.; Lee Y.-L.; Hsu C.-Y. Stathmin1 overexpression associated with polyploidy, tumor-cell invasion, early recurrence, and poor prognosis in human hepatoma. Mol. Carcinog. 2010, 49, 476–87. 10.1002/mc.20627. 20232364

[ref258] Belletti B.; Baldassarre G. Stathmin: a protein with many tasks. New biomarker and potential target in cancer. Expert Opin. Ther. Targets 2011, 15, 1249–66. 10.1517/14728222.2011.620951. 21978024

[ref259] Biaoxue R.; Xiguang C.; Hua L.; Shuanying Y. Stathmin-dependent molecular targeting therapy for malignant tumor: The latest 5 years’ discoveries and developments. J. Transl. Med. 2016, 14, 279 10.1186/s12967-016-1000-z. 27670291 PMC5037901

[ref260] Assali A.; Akhavan O.; Mottaghitalab F.; Adeli M.; Dinarvand R.; Razzazan S.; Arefian E.; Soleimani M.; Atyabi F. Cationic graphene oxide nanoplatform mediates miR-101 delivery to promote apoptosis by regulating autophagy and stress. Int. J. Nanomedicine 2018, 13, 5865–86. 10.2147/IJN.S162647. 30319254 PMC6171513

[ref261] Zhang L.; Lu Z.; Zhao Q.; Huang J.; Shen H.; Zhang Z. Enhanced chemotherapy efficacy by sequential delivery of siRNA and anticancer drugs using PEI-grafted graphene oxide. Small 2011, 7, 460–4. 10.1002/smll.201001522. 21360803

[ref262] Sabra S. A.; Elzoghby A. O.; Sheweita S. A.; Haroun M.; Helmy M. W.; Eldemellawy M. A.; Xia Y.; Goodale D.; Allan A. L.; Rohani S. Self-assembled amphiphilic zein-lactoferrin micelles for tumor targeted co-delivery of rapamycin and wogonin to breast cancer. Eur. J. Pharm. Biopharm. 2018, 128, 156–69. 10.1016/j.ejpb.2018.04.023. 29689288

[ref263] Maeda H.; Nakamura H.; Fang J. The EPR effect for macromolecular drug delivery to solid tumors: Improvement of tumor uptake, lowering of systemic toxicity, and distinct tumor imaging in vivo. Adv. Drug Delivery Rev. 2013, 65, 71–9. 10.1016/j.addr.2012.10.002. 23088862

[ref264] Hu C.; Gu F.; Gong C.; Xia Q.; Gao Y.; Gao S. Co-delivery of the autophagy inhibitor si-Beclin1 and the doxorubicin nano-delivery system for advanced prostate cancer treatment. J. Biomater. Appl. 2022, 36, 1317–31. 10.1177/08853282211060252. 34856824

[ref265] Zhang M.; Zhang W.; Tang G.; Wang H.; Wu M.; Yu W.; Zhou Z.; Mou Y.; Liu X. Targeted Codelivery of Docetaxel and Atg7 siRNA for Autophagy Inhibition and Pancreatic Cancer Treatment. ACS Appl. Bio Mater. 2019, 2, 1168–76. 10.1021/acsabm.8b00764. 35021365

[ref266] Gong C.; Hu C.; Gu F.; Xia Q.; Yao C.; Zhang L.; Qiang L.; Gao S.; Gao Y. Co-delivery of autophagy inhibitor ATG7 siRNA and docetaxel for breast cancer treatment. J. Controlled Release 2017, 266, 272–86. 10.1016/j.jconrel.2017.09.042. 28987884

[ref267] Thomson S. E.; Charalambous C.; Smith C. A.; Tsimbouri P. M.; Déjardin T.; Kingham P. J.; Hart A. M.; Riehle M. O. Microtopographical cues promote peripheral nerve regeneration via transient mTORC2 activation. Acta Biomater. 2017, 60, 220–31. 10.1016/j.actbio.2017.07.031. 28754648 PMC5593812

[ref268] Wang X.; Wu F.; Li G.; Zhang N.; Song X.; Zheng Y.; Gong C.; Han B.; He G. Lipid-modified cell-penetrating peptide-based self-assembly micelles for co-delivery of narciclasine and siULK1 in hepatocellular carcinoma therapy. Acta Biomater. 2018, 74, 414–29. 10.1016/j.actbio.2018.05.030. 29787814

[ref269] Nguyen H. T.; Tran T. H.; Thapa R. K.; Pham T. T.; Jeong J.-H.; Youn Y. S.; Choi H.-G.; Yong C. S.; Kim J. O. Incorporation of chemotherapeutic agent and photosensitizer in a low temperature-sensitive liposome for effective chemo-hyperthermic anticancer activity. Expert Opin. Drug Delivery 2017, 14, 155–64. 10.1080/17425247.2017.1266330. 27892715

[ref270] Jokerst J. V.; Lobovkina T.; Zare R. N.; Gambhir S. S. Nanoparticle PEGylation for imaging and therapy. Nanomedicine 2011, 6, 715–28. 10.2217/nnm.11.19. 21718180 PMC3217316

[ref271] Guan J.; Sun J.; Sun F.; Lou B.; Zhang D.; Mashayekhi V.; Sadeghi N.; Storm G.; Mastrobattista E.; He Z. Hypoxia-induced tumor cell resistance is overcome by synergistic GAPDH-siRNA and chemotherapy co-delivered by long-circulating and cationic-interior liposomes. Nanoscale 2017, 9, 9190–201. 10.1039/C7NR02663C. 28650490

[ref272] Lin Y. X.; Wang Y.; An H. W.; Qi B.; Wang J.; Wang L.; Shi J.; Mei L.; Wang H. Peptide-Based Autophagic Gene and Cisplatin Co-delivery Systems Enable Improved Chemotherapy Resistance. Nano Lett. 2019, 19, 2968–78. 10.1021/acs.nanolett.9b00083. 30924343

[ref273] Zhao P.; Li M.; Wang Y.; Chen Y.; He C.; Zhang X.; Yang T.; Lu Y.; You J.; Lee R. J.; Xiang G. Enhancing anti-tumor efficiency in hepatocellular carcinoma through the autophagy inhibition by miR-375/sorafenib in lipid-coated calcium carbonate nanoparticles. Acta Biomater. 2018, 72, 248–55. 10.1016/j.actbio.2018.03.022. 29555460

[ref274] Halamoda Kenzaoui B.; Chapuis Bernasconi C.; Guney-Ayra S.; Juillerat-Jeanneret L. Induction of oxidative stress, lysosome activation and autophagy by nanoparticles in human brain-derived endothelial cells. Biochem. J. 2012, 441, 813–21. 10.1042/BJ20111252. 22026563

[ref275] Hussain S.; Garantziotis S. Interplay between apoptotic and autophagy pathways after exposure to cerium dioxide nanoparticles in human monocytes. Autophagy 2013, 9, 101–3. 10.4161/auto.22266. 23047327 PMC3542208

[ref276] Fan J.; Sun Y.; Wang S.; Li Y.; Zeng X.; Cao Z.; Yang P.; Song P.; Wang Z.; Xian Z.; Gao H.; Chen Q.; Cui D.; Ju D. Inhibition of autophagy overcomes the nanotoxicity elicited by cadmium-based quantum dots. Biomaterials 2016, 78, 102–14. 10.1016/j.biomaterials.2015.11.029. 26686052

[ref277] Ou L.; Song B.; Liang H.; Liu J.; Feng X.; Deng B.; Sun T.; Shao L. Toxicity of graphene-family nanoparticles: A general review of the origins and mechanisms. Part. Fibre Toxicol. 2016, 13, 57 10.1186/s12989-016-0168-y. 27799056 PMC5088662

[ref278] Sun T.; Yan Y.; Zhao Y.; Guo F.; Jiang C. Copper oxide nanoparticles induce autophagic cell death in a549 cells. PLoS One 2012, 7, e43442 10.1371/journal.pone.0043442. 22916263 PMC3423358

[ref279] Chen G.-Y.; Yang H.-J.; Lu C.-H.; Chao Y.-C.; Hwang S.-M.; Chen C.-L.; Lo K.-W.; Sung L.-Y.; Luo W.-Y.; Tuan H.-Y.; Hu Y.-C. Simultaneous induction of autophagy and toll-like receptor signaling pathways by graphene oxide. Biomaterials 2012, 33, 6559–69. 10.1016/j.biomaterials.2012.05.064. 22704844

[ref280] Liu H. L.; Zhang Y. L.; Yang N.; Zhang Y. X.; Liu X. Q.; Li C. G.; Zhao Y.; Wang Y. G.; Zhang G. G.; Yang P.; Guo F.; Sun Y.; Jiang C. Y. A functionalized single-walled carbon nanotubeinduced autophagic cell death in human lung cells through Akt-TSC2-mTOR signaling. Cell Death Dis. 2011, 2, e159 10.1038/cddis.2011.27. 21593791 PMC3122114

[ref281] Li J. J.; Hartono D.; Ong C.-N.; Bay B.-H.; Yung L-Y L Autophagy and oxidative stress associated with gold nanoparticles. Biomaterials 2010, 31, 5996–6003. 10.1016/j.biomaterials.2010.04.014. 20466420

[ref282] Ma X.; Wu Y.; Jin S.; Tian Y.; Zhang X.; Zhao Y.; Yu L.; Liang X. J. Gold nanoparticles induce autophagosome accumulation through size-dependent nanoparticle uptake and lysosome impairment. ACS Nano 2011, 5, 8629–39. 10.1021/nn202155y. 21974862

[ref283] Johnson-Lyles D. N.; Peifley K.; Lockett S.; Neun B. W.; Hansen M.; Clogston J.; Stern S. T.; McNeil S. E. Fullerenol cytotoxicity in kidney cells is associated with cytoskeleton disruption, autophagic vacuole accumulation, and mitochondrial dysfunction. Toxicol. Appl. Pharmacol. 2010, 248, 249–58. 10.1016/j.taap.2010.08.008. 20713077 PMC2949473

[ref284] Lee Y. H.; Cheng F. Y.; Chiu H. W.; Tsai J. C.; Fang C. Y.; Chen C. W.; Wang Y. J. Cytotoxicity, oxidative stress, apoptosis and the autophagic effects of silver nanoparticles in mouse embryonic fibroblasts. Biomaterials 2014, 35, 4706–15. 10.1016/j.biomaterials.2014.02.021. 24630838

[ref285] Bramini M.; Sacchetti S.; Armirotti A.; Rocchi A.; Vazquez E.; Leon Castellanos V.; Bandiera T.; Cesca F.; Benfenati F. Graphene Oxide Nanosheets Disrupt Lipid Composition, Ca 2+ Homeostasis, and Synaptic Transmission in Primary Cortical Neurons. ACS Nano 2016, 10, 7154 10.1021/acsnano.6b03438. 27359048

[ref286] Wan B.; Wang Z. X.; Lv Q. Y.; Dong P. X.; Zhao L. X.; Yang Y.; Guo L. H. Single-walled carbon nanotubes and graphene oxides induce autophagosome accumulation and lysosome impairment in primarily cultured murine peritoneal macrophages. Toxicol. Lett. 2013, 221, 118–27. 10.1016/j.toxlet.2013.06.208. 23769962

[ref287] Orecna M.; De Paoli S. H.; Janouskova O.; Tegegn T. Z.; Filipova M.; Bonevich J. E.; Holada K.; Simak J. Toxicity of carboxylated carbon nanotubes in endothelial cells is attenuated by stimulation of the autophagic flux with the release of nanomaterial in autophagic vesicles. Nanomedicine Nanotechnology, Biol. Med. 2014, 10, e939 10.1016/j.nano.2014.02.001. 24566271

[ref288] Yu K. N.; Kim J. E.; Seo H. W.; Chae C.; Cho M. H. Differential toxic responses between pristine and functionalized multiwall nanotubes involve induction of autophagy accumulation in murine lung. J. Toxicol. Environ. Heal. - Part A Curr. Issues 2013, 76, 1282–92. 10.1080/15287394.2013.850137. 24283420

[ref289] Mittal S.; Sharma P. K.; Tiwari R.; Rayavarapu R. G.; Shankar J.; Chauhan L. K. S.; Pandey A. K. Impaired lysosomal activity mediated autophagic flux disruption by graphite carbon nanofibers induce apoptosis in human lung epithelial cells through oxidative stress and energetic impairment. Part. Fibre Toxicol. 2017, 14, 15 10.1186/s12989-017-0194-4. 28454554 PMC5408471

[ref290] Mishra A. R.; Zheng J.; Tang X.; Goering P. L. Silver nanoparticle-induced autophagic-Lysosomal disruption and NLRP3-inflammasome activation in HepG2 cells is size-dependent. Toxicol. Sci. 2016, 150, 473–87. 10.1093/toxsci/kfw011. 26801583

[ref291] Mao B. H.; Tsai J. C.; Chen C. W.; Yan S. J.; Wang Y. J. Mechanisms of silver nanoparticle-induced toxicity and important role of autophagy. Nanotoxicology 2016, 10, 1021–40. 10.1080/17435390.2016.1189614. 27240148

[ref292] Cohignac V.; Landry M. J.; Ridoux A.; Pinault M.; Annangi B.; Gerdil A.; Herlin-Boime N.; Mayne M.; Haruta M.; Codogno P.; Boczkowski J.; Pairon J. C.; Lanone S. Carbon nanotubes, but not spherical nanoparticles, block autophagy by a shape-related targeting of lysosomes in murine macrophages. Autophagy 2018, 14, 1323–34. 10.1080/15548627.2018.1474993. 29938576 PMC6103705

[ref293] Zhou H.; Gong X.; Lin H.; Chen H.; Huang D.; Li D.; Shan H.; Gao J. Gold nanoparticles impair autophagy flux through shape-dependent endocytosis and lysosomal dysfunction. J. Mater. Chem. B 2018, 6, 8127–36. 10.1039/C8TB02390E. 32254932

[ref294] Koga H.; Kaushik S.; Cuervo A. M. Altered lipid content inhibits autophagic vesicular fusion. FASEB J. 2010, 24, 3052–65. 10.1096/fj.09-144519. 20375270 PMC2909278

[ref295] Yang D. S.; Stavrides P.; Kumar A.; Jiang Y.; Mohan P. S.; Ohno M.; Dobrenis K.; Davidson C. D.; Saito M.; Pawlik M.; Huo C.; Walkley S. U.; Nixon R. A. Cyclodextrin has conflicting actions on autophagy flux in vivo in brains of normal and Alzheimer model mice. Hum. Mol. Genet. 2017, 26, 843–59. 10.1093/hmg/ddx001. 28062666 PMC6075207

[ref296] Kaushal N.; Tiruchinapally G.; Durmaz Y. Y.; Bao L.; Gilani R.; Merajver S. D.; ElSayed M. E. H. Synergistic inhibition of aggressive breast cancer cell migration and invasion by cytoplasmic delivery of anti-RhoC silencing RNA and presentation of EPPT1 peptide on “smart” particles. J. Controlled Release 2018, 289, 79–93. 10.1016/j.jconrel.2018.07.042. 30149048

[ref297] Durmaz Y. Y.; Lin Y.-L.; ElSayed M. E. H. Development of Degradable, pH-Sensitive Star Vectors for Enhancing the Cytoplasmic Delivery of Nucleic Acids. Adv. Funct. Mater. 2013, 23, 3885–95. 10.1002/adfm.201203762.

[ref298] Li X.; Xu H. L.; Liu Y. X.; An N.; Zhao S.; Bao J. K. Autophagy modulation as a target for anticancer drug discovery. Acta Pharmacol. Sin. 2013, 34, 612–24. 10.1038/aps.2013.23. 23564085 PMC4002878

[ref299] Yang Z. J.; Chee C. E.; Huang S.; Sinicrope F. A. Autophagy modulation for cancer therapy. Cancer Biol. Ther. 2011, 11, 169–76. 10.4161/cbt.11.2.14663. 21263212 PMC3230308

[ref300] López-Méndez T. B.; Sánchez-Álvarez M.; Trionfetti F.; Pedraz J. L.; Tripodi M.; Cordani M.; Strippoli R.; González-Valdivieso J. Nanomedicine for autophagy modulation in cancer therapy: a clinical perspective. Cell Biosci. 2023, 13, 104 10.1186/s13578-023-01057-9. 37296451 PMC10257261

